# CD98hc (SLC3A2) sustains amino acid and nucleotide availability for cell cycle progression

**DOI:** 10.1038/s41598-019-50547-9

**Published:** 2019-10-01

**Authors:** Sara Cano-Crespo, Josep Chillarón, Alexandra Junza, Gonzalo Fernández-Miranda, Judit García, Christine Polte, Laura R. de la Ballina, Zoya Ignatova, Óscar Yanes, Antonio Zorzano, Camille Stephan-Otto Attolini, Manuel Palacín

**Affiliations:** 1 Institute for Research in Biomedicine (IRB Barcelona), The Barcelona Institute of Science and Technology, Barcelona, 08028 Spain; 2 Department of Cell Biology, Physiology and Immunology, Faculty of Biology, Universitat de Barcelona, Barcelona, 08028 Spain; 3 Metabolomics Platform, IISPV, Department of Electronic Engineering (DEEEA), Universitat Rovira i Virgili, Tarragona, 43003 Spain; 4 Centro de Investigación Biomédica en Red de Diabetes y Enfermedades Metabólicas Asociadas (CIBERDEM), Madrid, 28029 Spain; 5 Secció Errors Congènits del Metabolisme-IBC, Servei de Bioquímica i Genètica Molecular, Hospital Clínic, Barcelona, Spain; 6 Centro de Investigación Biomédica en Red de Enfermedades Raras (CIBERER), Madrid, 28029 Spain; 7 Institute of Biochemistry and Molecular Biology University of Hamburg, Hamburg, 20246 Germany; 8 Department of Molecular Medicine, Institute of Basic Medical Sciences, Faculty of Medicine, University of Oslo, Oslo, 0372 Norway; 9 Centre for Cancer Cell Reprogramming, Institute for Clinical Medicine, Faculty of Medicine, University of Oslo, Oslo, 0372 Norway; 10 Department of Biochemistry and Molecular Biomedicine, Faculty of Biology, University of Barcelona, Barcelona, 08028 Spain

**Keywords:** DNA synthesis, Nutrient signalling, Cell-cycle exit, DNA synthesis, DNA damage and repair

## Abstract

CD98 heavy chain (CD98hc) forms heteromeric amino acid (AA) transporters by interacting with different light chains. Cancer cells overexpress CD98hc-transporters in order to meet their increased nutritional and antioxidant demands, since they provide branched-chain AA (BCAA) and aromatic AA (AAA) availability while protecting cells from oxidative stress. Here we show that BCAA and AAA shortage phenocopies the inhibition of mTORC1 signalling, protein synthesis and cell proliferation caused by CD98hc ablation. Furthermore, our data indicate that CD98hc sustains glucose uptake and glycolysis, and, as a consequence, the pentose phosphate pathway (PPP). Thus, loss of CD98hc triggers a dramatic reduction in the nucleotide pool, which leads to replicative stress in these cells, as evidenced by the enhanced DNA Damage Response (DDR), S-phase delay and diminished rate of mitosis, all recovered by nucleoside supplementation. In addition, proper BCAA and AAA availability sustains the expression of the enzyme ribonucleotide reductase. In this regard, BCAA and AAA shortage results in decreased content of deoxynucleotides that triggers replicative stress, also recovered by nucleoside supplementation. On the basis of our findings, we conclude that CD98hc plays a central role in AA and glucose cellular nutrition, redox homeostasis and nucleotide availability, all key for cell proliferation.

## Introduction

Amino acid (AA) transporters are pivotal for human physiology^1^. Heteromeric Amino Acid Transporters (HATs) are disulphide-bound heterodimers composed by a heavy subunit from the SLC3 family (CD98hc (SLC3A2) or rBAT (SLC3A1)) and a light subunit from the SLC7 family^2^. CD98hc-associated transporters have two well established roles^3^. CD98hc confers the complex the capacity to potentiate integrin-dependent signals via direct binding with cytoplasmic tails of β-integrin subunits^3–5^, while the light chain, which can be LAT1, LAT2, xCT, y^+^LAT1, y^+^LAT2 or asc1, mediates AA transport, conferring substrate specificity to the heterodimer^6^.

A growing body of evidence shows that the AA transport function of CD98hc plays a crucial role in the growth, proliferation, survival and metastasis of cancer cells^7,8^. Rapidly proliferating cells extensively reprogram metabolic pathways to meet their increased demand of AAs, which are used not only as building blocks for protein synthesis but also as nitrogen and carbon sources for the synthesis of nucleotides, amino sugars, and glutathione^9,10^. In this regard, cancer cells are able to upregulate their nutritional and antioxidant capacity by overexpressing the AA transporters CD98hc-LAT1 ((L)-type amino acid transporter 1) and CD98hc-xCT (cystine/glutamate transporter)^11,12^.

We previously studied the effects of the ablation of CD98hc from fibroblasts derived from embryonic stem cells that expressed LAT1-, xCT- and y^+^LAT2-CD98hc associated transporters^4,13^. CD98hc knock out (KO) fibroblasts failed to survive in standard culture conditions due to cell death by ferroptosis^13–15^. This phenomenon is attributed to the loss of CD98hc-xCT, a transporter that sustains cellular redox homeostasis by taking up cyst(e)ine, which is necessary for glutathione biosynthesis^16–18^. Although the addition of β-mercaptoethanol (β-ME) to the culture media rescued cell death, CD98hc KO cells still presented increased oxidative stress^13^. Moreover, these cells showed a shortage in the intracellular branched-chain AA (BCAA) and aromatic AA (AAA) content, which led to defective cell proliferation^13,19,20^. These results allowed us to establish that the AA transport function of CD98hc lies at the cross-road of oxidative and nutritional stress. However, the relative contribution of each stressor to the phenotype of CD98hc KO cells remained unknown.

Nutritional status regulates cell cycle progression in part by controlling protein synthesis via the mammalian target of rapamycin complex 1 (mTORC1)^21–24^. Moreover, nucleotide biosynthesis pathways have strict energetic and nutritional requirements. Indeed, *de novo* synthesis of purine and pyrimidine nucleotides relies on metabolic pathways that provide carbon and nitrogen precursors, including the AAs aspartate, glutamine, serine and glycine, as well as glucose and CO_2_. The major feeder pathways are glycolysis, the pentose phosphate pathway (PPP), the serine-glycine pathway, the tricarboxylic acid cycle and glutamine amidotransferase reactions^25^. Interestingly, BCAAs have been shown to constitute a potential alternative source of nitrogen for the synthesis of nucleotides^26^. Moreover, BCAAs can control glucose metabolism by regulating pyruvate dehydrogenase activity^27^, and like AAAs, can be shunted via anaplerosis to replenish the tricarboxylic acid cycle^28,29^. However, little attention has been devoted to the involvement of BCAA and AAA availability in nucleotide metabolism. Furthermore, CD98hc may also regulate glucose metabolism via direct interaction and stabilisation of Glucose transporter 1 (GLUT1)^30^. Given these observations, we hypothesised that CD98hc participates in the cellular nucleotide metabolism and therefore in cell cycle regulation, since nucleotide availability is tightly related to the adequacy of the progression of cell division^31,32^.

The data provided herein indicate that CD98hc supports the cellular nucleotide content, possibly by regulating glucose uptake and glycolysis, and, consequently, the activity of the PPP. In addition, BCAA and AAA availability has an impact on the reduction of ribonucleotides to the corresponding deoxynucleotides, thus balancing the cellular nucleotide pool. Our results highlight a novel role of CD98hc and proper BCAA and AAA availability in cell cycle regulation, since both are required for the maintenance of an adequate nucleotide pool for DNA synthesis, thereby protecting cells from DNA replication stress.

## Results

### BCAA and AAA shortage phenocopies part of the phenotype driven by CD98hc ablation: mTORC1 signalling downregulation without oxidative stress and eIF2α phosphorylation

**Figure 1:**
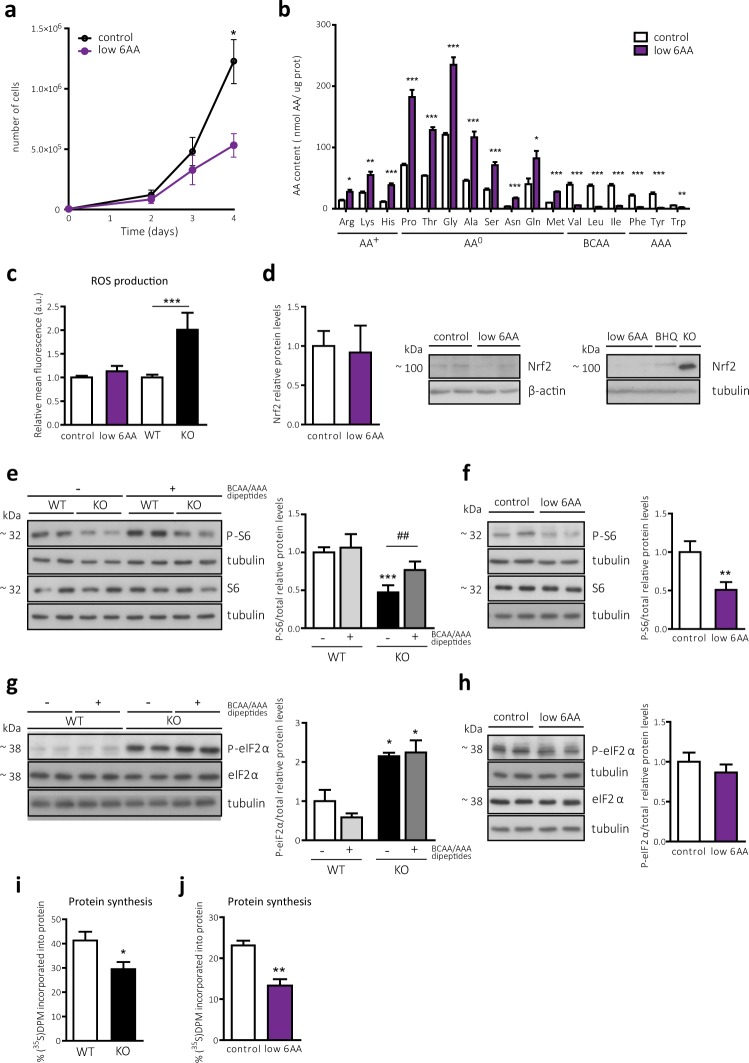
CD98hc ablation leads to BCAA and AAA shortage-dependent mTORC1 inactivation and –independent eIF2-mediated integrated stress response with reduced general protein synthesis. (**a**) Proliferation of control and low 6AA cells. n = 3. (**b**) Intracellular AA content in control and low 6AA cells. AAs are grouped by side chain properties as indicated. n = 5. (**c**) ROS levels quantified by flow cytometry using the fluorescent free radical sensor H_2_DCFDA in control, low 6AA, WT and CD98hc KO cells. a.u., arbitrary units. n = 3. (**d**) Nrf2 protein expression in control and low 6AA cells (left panels). n = 4. Tert-butylhydroquinone (BHQ) treatment (50 µM, 1 h) and CD98 KO cells were used as positive controls of oxidative stress (right panel, representative experiment). 50 μg of protein extracts were loaded in each lane. Data are normalised by β-actin and tubulin expression (left and right panels, respectively). Full-length blots are presented in Supplementary Fig. S2. (**e**) S6 phosphorylation in WT and CD98hc KO cells with no additives or in the presence of BCAA- and AAA- containing dipeptides. Data are normalised by total levels of S6 protein and tubulin expression. n = 4. Full-length blots are presented in Supplementary Fig. S2. (**f**) S6 phosphorylation in control and low 6AA cells. Data are normalised by total levels of S6 protein and tubulin expression. n = 4. Full-length blots are presented in Supplementary Fig. S2. (**g**) eIF2α phosphorylation in WT and CD98hc KO cells with no additives or in the presence of BCAA- and AAA- containing dipeptides. Data are normalised by total levels of eIF2α protein and tubulin expression. n = 3. Full-length blots are presented in Supplementary Fig. S2**. (h**) eIF2α phosphorylation in control and low 6AA cells. Data are normalised by total levels of eIF2α protein and tubulin expression. n = 3. Full-length blots are presented in Supplementary Fig. S2. (**i**,**j**) ^35^S-methionine incorporation into protein in WT and CD98hc KO cells (**i**) and control and low 6AA cells (**j**). DPM, disintegrations per minute. n = 4. Data quantification correspond to the mean ± SEM of the independent experiments (n) indicated for each graph normalised to control or WT cells. Statistical significance *p ≤ 0.05; **p ≤ 0.01; ***p ≤ 0.001 vs. control or WT cells, ^#^p ≤ 0.05; ^##^p ≤ 0.01; ^###^p ≤ 0.001 vs. CD98hc KO cells was analysed using a Student’s t‐test (panels a, b, c, i and j) or a linear model (panels d, e, f, g and h).

Fibroblasts derived from embryonic stem cells lacking CD98hc-related transporters showed a shortage of BCAAs and AAAs and increased reactive oxygen species (ROS)^13^. In order to dissociate oxidative from nutritional stress, we generated a cellular model with only one of the stressors. To this end, we cultured wild-type (WT) cells in media with reduced concentrations of BCAAs and AAAs, considered within the lower physiological levels in plasma (Supplementary Fig. S1), under standard cell culture concentrations of cyst(e)ine and β-ME. Cell culture medium was optimised to phenocopy the proliferation defect (Fig. 1a) reported in the CD98hc KO model^13^. These cells (hereafter referred to as low 6AA cells) showed a dramatic decrease in the content of BCAAs and AAAs compared with those cultured in complete media (control cells) (Fig. 1b). Strikingly, the intracellular levels of cationic (AA^+^) and neutral (AA^0^) AAs were increased in low 6AA cells (Fig. 1b). This imbalance in the intracellular AA content (Supplementary Fig. S1) resembled that observed in CD98hc KO cells^13^. The alteration in the expression of other transporters in low 6AA cells may account for the increase in the AA^+^ concentration^13^, as indicated by higher mRNA expression levels of the AA^+^ transporters CAT1 and CAT3 (y^+^ transport system) and y^+^LAT1 (y^+^L transport system) in these cells (Supplementary Fig. S1). This finding is consistent with increased L-arginine uptake by both the y^+^ and y^+^L transport systems in low 6AA cells (Supplementary Fig. S1). In the light of these results, extracellular BCAA and AAA restriction is sufficient to trigger the intracellular imbalance of AAs observed in cells lacking CD98hc. Oxidative stress was not increased in low 6AA cells, since no differences were found compared to control cells in the redox-sensitive H_2_DCFDA labelling (Fig. 1c). In addition, the protein levels of Nuclear factor (erythroid-derived 2)-like 2 (Nrf2), considered a master regulator of intracellular antioxidant response^33^, were not increased in low 6AA compared to control cells (Fig. 1d). This observation indicates that in contrast to CD98hc KO cells (Fig. 1c,d), low 6AA cells did not present oxidative stress. Together, these findings point to low 6AA cells as a suitable cellular model in which to study the effects of cellular BCAA and AAA deficiency, independently of oxidative stress and other possible metabolic alterations that might be associated with CD98hc ablation.

The nutrient-sensing pathway mTORC1 responds to AA deprivation by downregulating global protein synthesis while reprograming cells for their particular needs^34^. mTORC1 activation stimulates the subsequent phosphorylation of components of the translational machinery, including the ribosomal protein S6, one of the most widely studied downstream effector targets of this pathway^35^. Consistent with the AA shortage, CD98hc KO cells showed lower levels of phosphorylated S6 (P-S6) in comparison to WT cells (Fig. 1e), in agreement with the alterations reported in cells lacking CD98hc-LAT1^8,19,36^. The addition of BCAA- and AAA-containing dipeptides to the culture media, which can be transported by CD98hc KO cells via the dipeptide transporter 1 (PEPT1)^13^, partially restored S6 phosphorylation (Fig. 1e). In addition, low 6AA cells also presented repressed mTORC1 activation, as revealed by decreased P-S6 protein levels (Fig. 1f). These results indicate that the shortage of BCAAs and AAAs in CD98hc KO cells is responsible of the downregulation of mTORC1 pathway, which may repress protein synthesis in these cells.

AA deprivation, among other stress stimuli, leads to the activation of the eIF2-mediated integrated stress response^37,38^. eIF2α phosphorylation reduces the overall rate of translation, allowing cells to overcome the stress or promoting their elimination if the damage cannot be repaired^39^. In line with previous results^13^, CD98hc KO cells presented a marked increase in the phosphorylated levels of eIF2α (P-eIF2α) compared to WT cells (Fig. 1g). Interestingly, the levels of P-eIF2α did not change after addition of BCAA- and AAA-containing dipeptides (Fig. 1g), thereby suggesting that nutritional status was not the trigger of the eIF2α-mediated integrated stress response pathway activation in CD98hc KO cells. The phosphorylation of the α subunit of eIF2 caused by AA deprivation is mediated by the kinase general control non-derepressible-2 (GCN2)^40^, which is activated through the binding of uncharged transfer RNAs (tRNAs)^41^. Importantly, tRNA charging levels, measured by the tRNA-tailored microarrays, were only marginally affected in CD98hc KO cells (Supplementary Fig. S1), which suggests that GCN2 is not upstream of eIF2α phosphorylation in these cells. Moreover, phosphorylated levels of eIF2α remained unaffected in low 6AA cells when compared to control cells (Fig. 1h), which reinforces the notion that the activation of the integrated stress response mediated by eIF2 is unlikely to be related to the shortage of BCAAs and AAAs that resulted from CD98hc ablation.

Consistent with the alterations in the two signalling pathways, general protein synthesis was decreased in cells lacking CD98hc, as evidenced by the lower [^35^S]-methionine incorporation into newly synthesized proteins (Fig. 1i). Given that low 6AA cells presented a similar downregulation in protein translation (Fig. 1j), mTORC1 downregulation is likely to occur upstream of repressed protein synthesis in CD98hc KO cells. However, the impact of P-eIF2α cannot be discarded.

### Transcriptome analysis reveals putative cell cycle alterations in CD98hc KO cells

**Figure 2:**
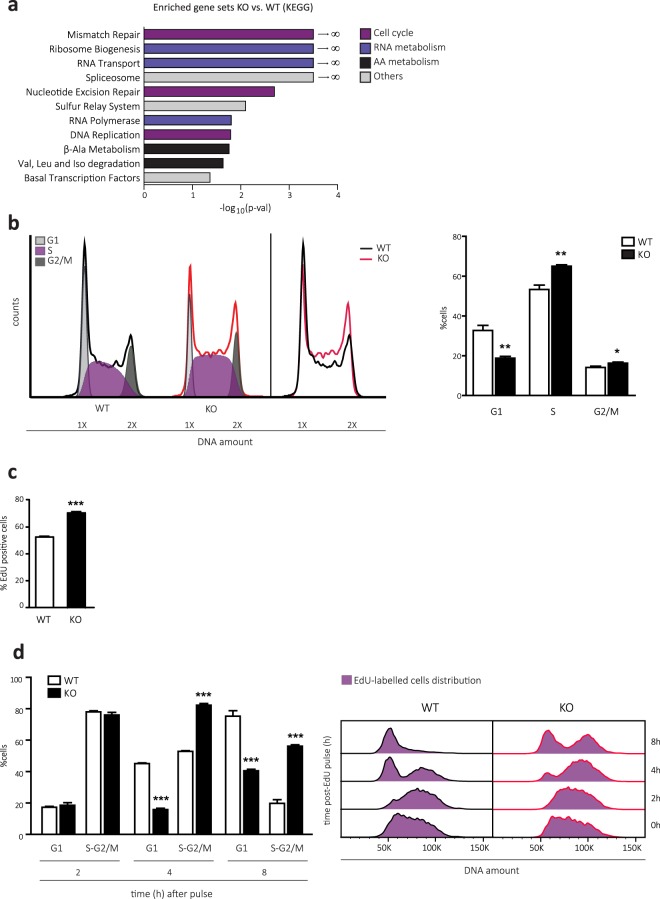
CD98hc depletion leads to delayed S-phase. (**a**) Gene Set Enrichment Analysis (GSEA) of transcriptional data from WT and CD98hc KO cells. Bars represent significantly positively enriched gene sets (nominal p-value < 5%, FDR < 25%) in CD98hc KO cells compared to WT cells according to the KEGG data base. Bars are coloured following the functional characterisation as indicated. X-axis: -log_10_ (p-val). (**b**) Cell cycle distribution measured by flow cytometry using propidium iodide (PI) staining. 10,000 cells/condition were analysed. A representative cell cycle profile of WT and CD98hc KO cells is shown, along with the overlap of their profiles (left panel). The graphical representation of cell cycle distribution (right panel) shows the percentage of cells in G1, S and G2/M phases in WT and CD98hc KO cells. n = 4. (**c**) Percentage of EdU-positive cells. WT and CD98hc KO cells were pulsed with 10 μM EdU for 1 h and EdU incorporation was quantified by FACS from the corresponding gates displayed in Supplementary Fig. S4 at 0 h. 10,000 cells/condition were analysed. n = 3. (**d**) EdU pulse-chase time course showing the dynamics WT and CD98hc KO cells over 8 h. Cells were pulsed with 10 μM EdU for 1 h and stained with propidium iodide (PI) and fluorescent azide. EdU incorporation and PI staining were quantified by FACS. Left panel, the percentage of Edu-positive WT and CD98hc KO cells in G1 and S-G2/M phases out of total was quantified from the corresponding gates displayed in Supplementary Fig. S4 at the indicated time points. 10,000 cells/condition were analysed. n = 3. Right panel, representative histogram overlay plot of DNA (PI) from the EdU-positive cells over varying time points. Data quantification correspond to the mean ± SEM of the independent experiments (n) indicated for each graph. Statistical significance *p ≤ 0.05; **p ≤ 0.01; ***p ≤ 0.001 vs. WT cells was analysed using a Student’s t‐test.

Besides protein synthesis, other cellular processes are known to be regulated by the nutritional status of the cell. In order to study additional alterations that could take place in cells surviving loss of CD98hc, and, by consequence, their associated transport activities and AA shortage, a comparative transcriptome analysis was performed in CD98hc KO and WT cells. Strikingly, we found that 20% of the genes were differentially expressed in both groups (biological fold change > 2 and adjusted p-value < 0.05; GEO accession code: GSE126781). Supporting this, Gene Set Enrichment Analysis (GSEA) using KEGG database identified several gene categories altered between WT and CD98hc KO cells. Remarkably, we found a notable enrichment in gene sets closely related to the cell cycle, including mismatch repair, DNA replication and nucleotide excision repair (Fig. 2a and Supplementary Fig. S3; GEO accession code: GSE126781). Furthermore, gene sets associated with RNA and AA metabolism were also enriched in CD98hc KO cells, which may be caused by BCAA and AAA deficiency and defective protein synthesis (Fig. 2a and Supplementary Fig. S3; GEO accession code: GSE126781).

### Cells lacking CD98hc fail to progress adequately through the S-phase of the cell cycle

On the basis of this transcriptome analysis, we analysed cell cycle phase distributions by measuring DNA content using flow cytometry in CD98hc KO and WT cells (Fig. 2b). Compared to WT, CD98hc KO cells showed increased S- (65 ± 0.7% vs. 53.3 ± 2.2%) and G2/M-phases (16 ± 0.5% vs. 14 ± 0.6%) at the expense of a reduction in G1-phase (18.8 ± 0.9% vs. 32.7 ± 2.5%) (Fig. 2b). The increase in the S-phase population cannot be attributed to an enhanced proliferative rate, since cells lacking CD98hc presented a major delay in proliferation in comparison to WT cells^13^. Alternatively, the lack of CD98hc may slow down the progression of cells that are in the S-phase. To test whether CD98hc KO cells presented delayed DNA replication, they were pulsed for 1 h with 5-ethynyl-2′-deoxyuridine (EdU) to detect and quantify active DNA synthesis^42^. CD98hc KO cells showed increased EdU labelling compared to WT cells (Fig. 2c and Supplementary Fig. S4; time 0 h), thereby reinforcing our previous result (Fig. 2b). The progression of the formerly EdU labelled cells through the cell cycle phases was then monitored (Fig. 2d, left panel). Remarkably, at 4 h post-EdU pulse, 82.3 ± 1.1% of labelled CD98hc KO cells remained in S-G2/M phases as compared to 52.9 ± 0.4% in the case of WT cells, of which 45.1 ± 0.5% had divided and progressed to G1, in sharp contrast to the 15.8 ± 0.8% CD98hc KO cells (Fig. 2d, left panel, and Supplementary Fig. S4). This delay was evidenced over time as shown in the histogram overlay, corresponding to a representative experiment (Fig. 2d, right panel). To corroborate this finding, cells were synchronised in S-phase with a double block of thymidine^43^. The treatment achieved the retention of around 75% of cells in the DNA-synthesis phase, and S-phase progression was then monitored (Supplementary Fig. S4). Similar results were obtained with this approach, thereby confirming that cells lacking CD98hc fail to progress adequately through the DNA synthesis phase.

### BCAA and AAA limitation reproduces the replicative stress observed in CD98hc KO cells

**Figure 3:**
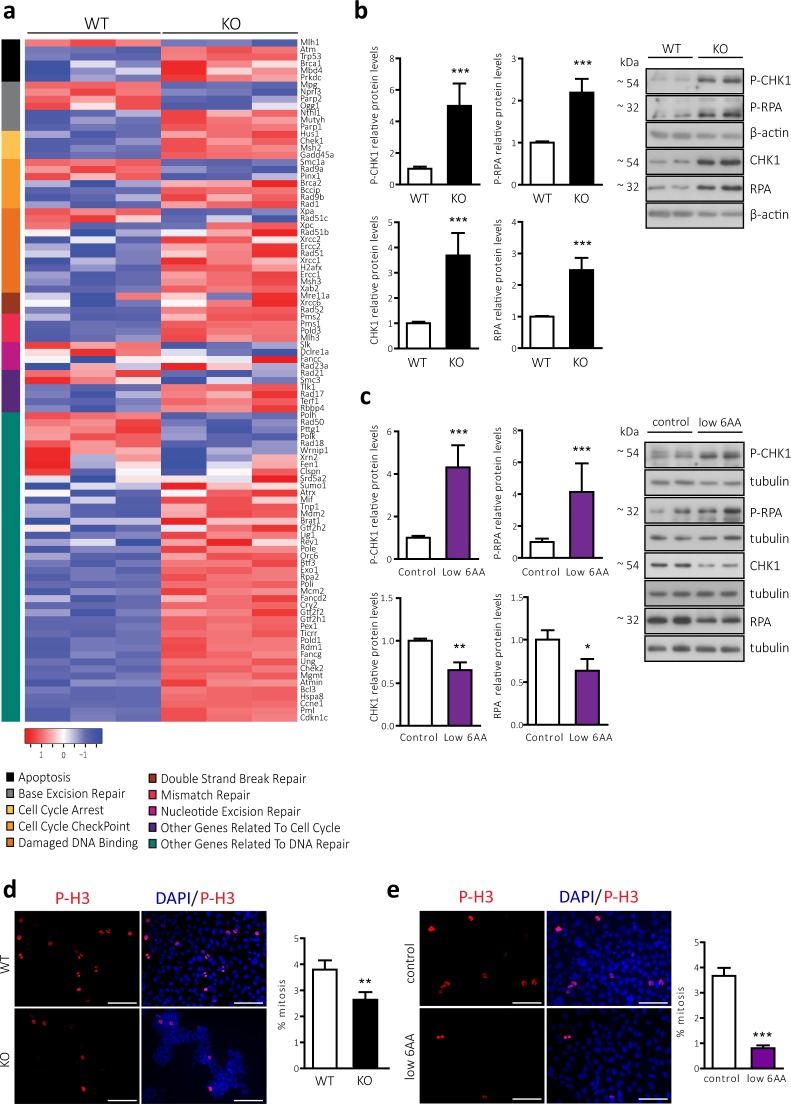
CD98hc deletion and BCAA and AAA shortage lead to replicative stress and impaired mitotic rate (**a**) Heat map of RNA expression level of 97 genes involved in DNA damage signalling pathways (see *Methods*) in WT and CD98hc KO cells grouped in the specified categories. Rows: genes; columns: samples. Range of colours (red/high to blue/low) shows the range of expression values after scaling within each sample. (**b**) Phosphorylated and total protein levels of CHK1 an RPA in WT and CD98hc KO cells. Data are normalised by β-actin expression. n = 5. Full-length blots are presented in Supplementary Fig. S6. (**c**) Phosphorylated and total protein levels of CHK1 an RPA in control and low 6AA cells. Data are normalised by tubulin expression. n = 3. Full-length blots are presented in Supplementary Figs. S6 and S2 (lowest panel, tubulin corresponding to CHK1). (**d**) Mitotic rate in WT and CD98hc KO by immunofluorescence. The phosphorylation of Histone H3 (P-H3, red) was used as a marker for cells undergoing mitosis. DNA was stained with DAPI (blue) (left panel). Scale bar is 50 microns. More than 30,000 nuclei/condition were analysed. The percentage of mitotic cells is shown (right panel). n = 7. (**e**) Mitotic rate in control and low 6AA cells by immunofluorescence. The phosphorylation of Histone H3 (P-H3, red) was used as a marker for cells undergoing mitosis. DNA was stained with DAPI (blue) (left panel). Scale bar is 50 microns. More than 30,000 nuclei/condition were analysed. The percentage of mitotic cells is shown (right panel). n = 7. Data quantification correspond to the mean ± SEM of the independent experiments (n) indicated for each graph normalised to WT or control cells. Statistical significance *p ≤ 0.05; **p ≤ 0.01; ***p ≤ 0.001 vs. WT or control cells was analysed using a linear model (panels b and c) or a Student’s t‐test (panels d and e).

During S-phase, cells must faithfully duplicate their genomes. As a consequence of DNA damage, cells suffer DNA replicative stress, which is characterised by the activation of the DNA damage response (DDR) pathway and often accompanied by cell cycle arrest^44,45^. We took advantage of the transcriptome analysis performed in WT and CD98hc KO cells to further interrogate whether the observed cell cycle arrest was accompanied by DNA damage and replicative stress in CD98hc KO cells. For this purpose, we created the DDR gene set, which comprised 97 genes related to the regulation of DNA replication and repair and involved in DNA damage signalling pathways. The selection was carried out following the bibliography and available gene lists from commercial arrays (RT² Profiler™ PCR Array Human DNA Repair (PAHS-042Z), QUIAGEN;)^46,47^. This gene set was significantly enriched in CD98hc KO cells (p-val < 0.001, NES = 1.83, FDR < 0.001) (Fig. 3a and Supplementary Fig. S5).

Replicative stress usually results in the development of stretches of single-stranded DNA rapidly coated by replication protein A (RPA), which functions as a signalling platform to recruit a wide variety of proteins involved in the DDR pathway^48^. The phosphorylation of RPA and checkpoint kinase 1 (CHK1), the final transducer of this signalling pathway, is widely accepted as the most specific indicator of DDR activation^49–51^. CD98hc KO cells presented enhanced DDR as demonstrated by the increased in phosphorylated and total levels of CHK1 and RPA (Fig. 3b), in line with the transcriptomic analysis (Fig. 3a). The overexpression of these two DDR markers is probably the outcome of an adaptation, which ensures that CD98hc KO cells survive in a context of chronic replicative stress. This phenomenon, which is also observed in tumour cells subjected to chronic replicative stress imposed by the tumour microenvironment^52–57^, along with the enhanced activation of the DDR, suggests that CD98hc ablation makes cells heavily reliant on this pathway in order to guarantee survival. Consistent with this assumption, AZD7762 treatment, which is a potent and selective inhibitor of CHK1^58^, significantly induced mortality in cells lacking CD98hc, whereas little impact was detected in WT cells (Supplementary Fig. S5).

Furthermore, apoptosis is often associated with inappropriate DNA damage resolution^59^. In this regard, CD98hc KO cells presented increased apoptotic cell death (Supplementary Fig. S5), which was already indicated by the results of the transcriptome analysis (Fig. 3a).

To address whether BCAA and AAA limitation poses a challenge for the integrity of DNA replication in CD98hc KO cells, we evaluated the same DDR indicators in low 6AA cells. CHK1 and RPA phosphorylation was strongly upregulated in low 6AA cells compared to control cells (Fig. 3c). In this case, the total levels of both proteins were decreased in low 6AA cells (Fig. 3c). The observed coupling between activating phosphorylation and subsequent downregulation of total levels of CHK1 has been described as a mechanism through which detrimental accumulation of this protein upon acute induction of genotoxic stresses is prevented^60^. Taken together, these results indicate that loss of CD98hc and shortage of BCAAs and AAAs compromise the cell cycle by triggering DNA damage and replicative stress.

### CD98hc and BCAA/AAA availability are required for correct completion of the cell division cycle

A key role of CHK1 within the DNA surveillance program is to stop cells from undergoing mitosis, thereby preventing the propagation of error-containing copies of the genome to daughter cells^61^. In this regard, we found an increase in the expression of genes involved in the transition from G2-phase to mitosis in CD98hc KO cells, according to the Hallmark database (Supplementary Fig. S5). This observation suggests that the entry into mitosis is compromised by the activation of the CHK1-mediated G2/M checkpoint. Mitosis was analysed by immunofluorescence using the marker phospho-histone H3 (P-H3). Specific phosphorylation at serine 10 (Ser10) of H3 starts during late G2-phase and peaks during mitosis, when it undergoes different localization patterns during the mitotic phases, depending on chromatin condensation^62,63^. Thus, only patterns corresponding to cells undergoing mitosis were selected for quantification. CD98hc KO cells showed an impaired mitotic rate compared to WT cells (2.64% ± 0.3 vs. 3.8% ± 0.4%) (Fig. 3d). This outcome was consistent with the increase in the percentage of CD98hc KO cells in the G2/M fraction (Fig. 2b), which may reflect a delay in the G2-phase caused by mitotic blockage^64^. Mitotic activity was also strongly diminished in low 6AA cells (0.80 ± 0.1% vs. 3.67 ± 0.3%) (Fig. 3e), thereby indicating that BCAA and AAA availability is essential for cell division.

### CD98hc KO cells present a defective pentose phosphate pathway and a general reduction in nucleotide pool levels

**Figure 4:**
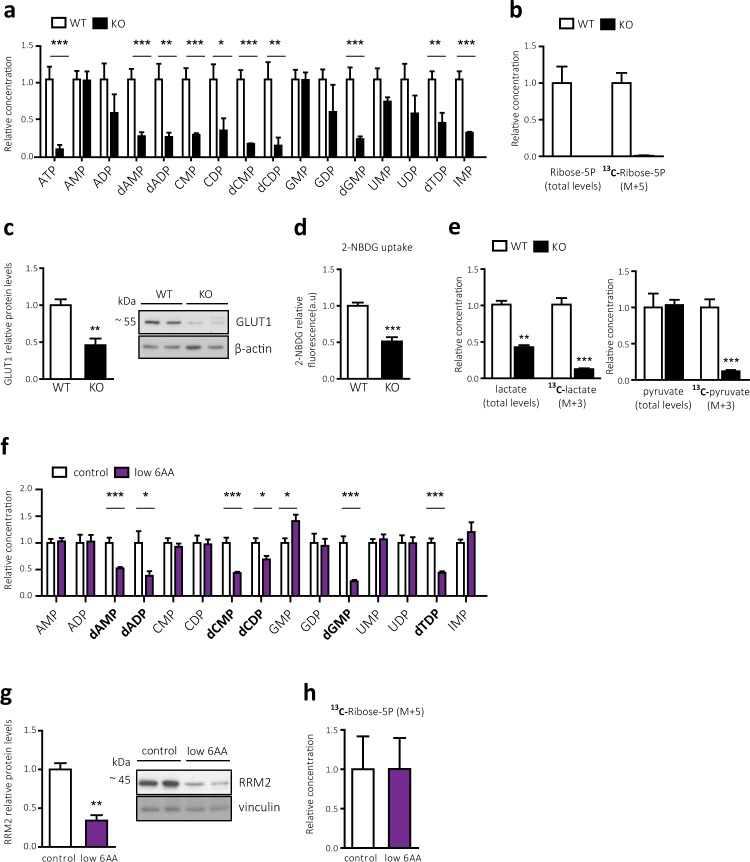
CD98hc and BCAA and AAA availability are required for correct maintenance of the intracellular nucleotide pool (**a**) Content of nucleotides in WT and CD98hc KO cells. Data are normalized to cell number. n = 5. (**b**) PPP activity was analysed by stable isotope tracer-based metabolomics in WT and CD98 KO cells. Total content of ribose-5P and ^13^C-ribose-5P (M + 5; five ^13^C carbon atoms) in WT and CD98hc KO cells. Data are normalized to cell number. n = 5. (**c**) GLUT1 protein expression in total membranes of WT and CD98hc KO cells. Data are normalized by β-actin expression. n = 4. Full-length blots are presented in Supplementary Fig. S7**. (d**) Glucose uptake measured by using the fluorescent glucose analogue 2-NBDG in WT and CD98hc KO cells. a.u., arbitrary units. n = 4. (**e**) Glycolysis analysed by stable isotope tracer-based metabolomics in WT and CD98hc KO cells. Total content of lactate and ^13^C-lactate (M + 3; three ^13^C carbon atoms) (left panel) and total content of pyruvate and ^13^C-pyruvate (M + 3; three ^13^C carbon atoms) (right panel) in CD98hc KO and WT cells. n = 5. (**f**) Content of nucleotides in control and low 6AA cells. Deoxynucleotides are highlighted. Data are normalized to cell number. n = 5. (**g**) RRM2 protein expression in control and low 6AA cells. Data are normalised by vinculin expression. n = 4. Full-length blots are presented in Supplementary Fig. S7**. (h**) PPP activity was analysed by stable isotope tracer-based metabolomics in control and low 6AA cells. ^13^C-Ribose-5P (M + 5; five ^13^C carbon atoms) in control and low 6AA cells. n = 5. For isotope tracer-based metabolomics analysis (**b**,**e**,**h**) cells were cultured with 5 mM fully labelled glucose (U-13C6-Glucose) for 16 h. Data quantification correspond to the mean ± SEM of the independent experiments (n) indicated for each graph normalised to WT or control cells. Statistical significance *p ≤ 0.05; **p ≤ 0.01; ***p ≤ 0.001 vs. WT or control cells was analysed using a Student’s t‐test (panels a, b, d, e, f and h) or a linear model (panels c and g).

To gain further insight into the link between the lack of CD98hc and the reported DNA damage, we performed a targeted metabolomics assay to quantify the nucleotide content of these cells. Notably, CD98hc KO cells showed a remarkable general decrease in nucleotide levels when compared to WT cells (Fig. 4a). This finding suggests that the alteration underlying this massive reduction compromised the biosynthesis of both purine and pyrimidine nucleotides before the formation of their precursors (Fig. 4a). Ribose-5-phosphate (5P), which is both a product and an intermediate of the PPP, plays a critical role in *de novo* nucleic acid synthesis. In this regard, ribose-5P functions as the scaffold for purine biosynthesis, but it also conforms the five-carbon sugar molecule of both purine and pyrimidine ribonucleotides^65^. In order to analyse the activity of the PPP, we used fully labelled glucose (U-^13^C6-glucose) in combination with gas chromatography-coupled to mass spectrometry. The PPP flux was abrogated in CD98hc KO cells in comparison to WT cells, as indicated by the absence of ^13^C-Ribose-5P (M + 5, five ^13^C‐labelled carbons) (Fig. 4b). In addition, total levels of ribose-5P were measured, and accordingly, they were prominently decreased in the CD98hc KO cells (Fig. 4b). Taken together, these results suggest that CD98hc KO cells present an impaired nucleotide synthesis as a result of reduced ribose-5P production, which is attributable to a decrease in the PPP flux.

### CD98hc sustains cellular glucose uptake and glycolysis independently of AA availability

After uptake, glucose is broken down to extract energy through the glycolysis pathway and it is also shunted to fuel the PPP. To determine whether compromised glucose uptake and disposal underlies the repressed PPP, we examined GLUT1 expression. Supporting previous reported results^30^, CD98hc KO cells showed a marked downregulation of GLUT1 protein expression (Fig. 4c), paralleled with a decreased glucose uptake in comparison to WT cells (Fig. 4d). To examine the contribution of glucose to the glycolytic pathway, we analysed the incorporation of ^13^C-glucose into the M + 3 (three ^13^C-labelled carbons) isotopologues ^13^C-pyruvate and ^13^C-lactate. In keeping with the decreased glucose uptake, lack of CD98hc depressed glycolysis, as evidenced by the lower incorporation of ^13^C-glucose into both metabolites in cells lacking CD98hc compared to WT cells (Fig. 4e). The content of unlabeled lactate was also diminished in the former cells. However, although less incorporation of ^13^C-glucose into ^13^C-pyruvate was detected in CD98hc KO cells, no changes were found regarding total levels of this metabolite, thereby pointing to an alternative source for its production (e.g., from extracellular 1 mM pyruvate) (Fig. 4e). Collectively, these results indicate that the defective glycolytic capacity of CD98hc KO cells is likely to underlie the compromised PPP, which ultimately results in nucleotide scarcity and replicative stress. In addition, the abrogated PPP activity in CD98hc KO cells can be potentiated by reduced expression of glucose-6-phosphate dehydrogenase (G6PDH) mRNA (Supplementary Fig. S5), which catalyses the rate-limiting step in the oxidative branch of the PPP^66^.

We next examined whether low 6AA cells also presented same alterations in nucleotide metabolism. Interestingly, in this case we only found a reduction in the deoxynucleotide content (Fig. 4f). This result suggests that low 6AA cells present an impaired conversion of nucleotides to deoxynucleotides. The ribonucleotide reductase is the only enzyme able to catalyse this rate-limiting step^67^. Its activity is determined by the levels of its ribonucleotide reductase regulatory subunit M2 (RRM2)^68^. Protein levels of RRM2 were found to be strongly diminished in low 6AA compared to control cells (Fig. 4g). As expected, PPP activity was not affected in low 6AA cells as indicated by levels of ^13^C-ribose-5P (M + 5, five ^13^C-labelled carbons) (Fig. 4h). These results strongly suggest that the decrease in deoxynucleotide levels is caused by suppression of RRM2 expression in low 6AA cells.

### Shortage of nucleotides causes replicative stress in CD98hc KO cells

**Figure 5:**
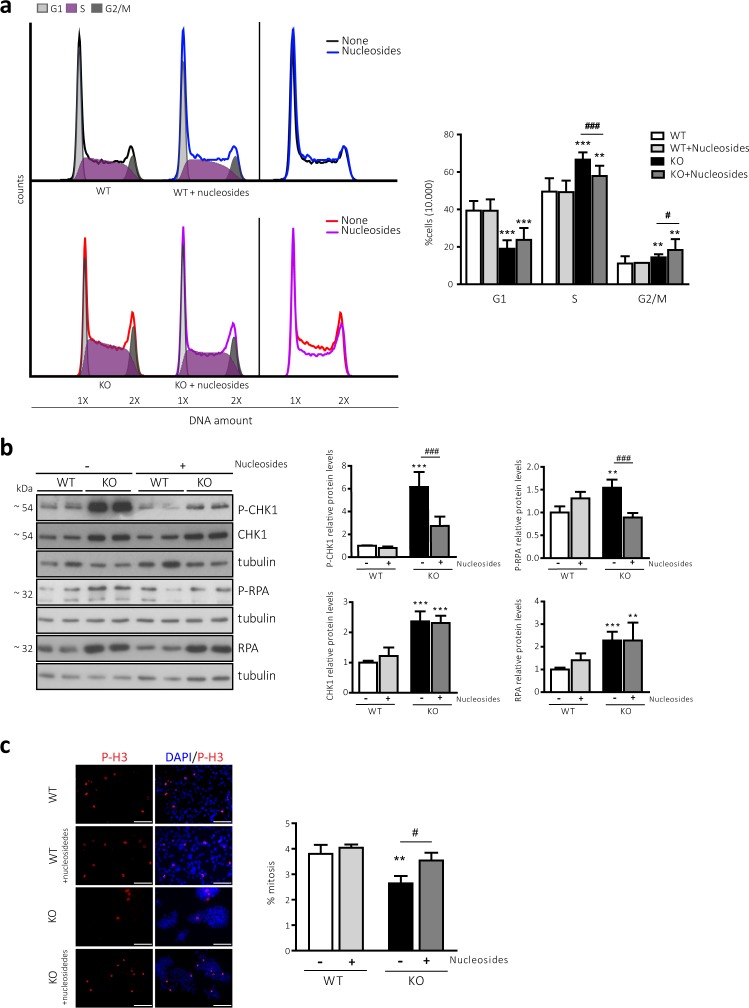
Nucleosides reverse cell cycle alterations in CD98hc KO cells. (**a**) Cell cycle distribution was measured by flow cytometry using propidium iodide (PI) staining. A representative cell cycle profile of WT and CD98hc KO cells with no additives or in the presence of nucleosides (150 µM cytidine, 150 µM guanosine, 150 µM uridine, 150 µM adenosine and 50 µM thymidine for 48 h) is shown, along with the overlap of their profiles (left panel). The graphical representation of cell cycle distribution shows the percentage of cells in G1, S and G2/M phases (right panel). n = 4. (**b**) Phosphorylated and total protein levels of CHK1 (n = 6) and RPA (n = 4) in WT and CD98hc KO cells with no additives or in the presence of nucleosides (150 µM cytidine, 150 µM guanosine, 150 µM uridine, 150 µM adenosine and 50 µM thymidine for 48 h). Data are normalised by tubulin expression. Full-length blots are presented in Supplementary Fig. S8**. (c**) Mitotic rate in WT and CD98hc KO cells with no additives or in the presence of nucleosides (150 µM cytidine, 150 µM guanosine, 150 µM uridine, 150 µM adenosine and 50 µM thymidine for 48 h) by immunofluorescence. The phosphorylation of Histone H3 (P-H3, red) was used as a marker for cells undergoing mitosis. DNA was stained with DAPI (blue) (left panel). Scale bar is 50 microns. Quantification of the percentage of mitotic cells is shown. More than 30,000 nuclei/condition from six independent experiments were analysed (right panel). Data quantification correspond to the mean ± SEM of the independent experiments (n) indicated for each graph normalised to WT cells. Statistical significance *p ≤ 0.05; **p ≤ 0.01; ***p ≤ 0.001 vs. WT or ^#^p ≤ 0.05; ^##^p ≤ 0.01; ^###^p ≤ 0.001 vs. CD98hc KO cells was analysed using a Student’s t-test (panels a and c) or a linear model (panel b).

We next sought to assess whether the decrease in nucleotides was responsible for the replicative stress in CD98hc KO cells. To test this hypothesis, we examined whether supplementation of nucleosides in the culture media could rescue S-phase delay in these cells. Hence, cell culture media was supplemented with the five (A, U, C, G and T) nucleosides, and cell cycle distribution was evaluated after 48 h. The percentage of cells that remained in the S fraction after nucleoside addition decreased from 66.6 ± 3.8% to 57.8 ± 5.4% in CD98hc KO cells (Fig. 5a), whereas the addition of nucleosides did not change cell cycle distribution in WT cells (Fig. 5a). This observation indicates that the shortage of nucleosides poses a replication barrier that delays the S-phase transition in CD98hc KO cells. To further corroborate our hypothesis, we next studied the effects of exogenous nucleosides on the activation of the DDR signalling pathway in CD98hc KO cells. The phosphorylation of CHK1 and RPA was strongly reduced in CD98hc KO cells supplemented with exogenous nucleosides, compared to non-treated cells; while the total levels of the two proteins remained unchanged after supplementation (Fig. 5b). Finally, we examined the effects of nucleoside addition on progression through the cell division cycle. To this end, we used P-H3-labelling by immunofluorescence in order to select and quantify cells undergoing mitosis. The mitotic rate was significantly recovered in nucleoside supplemented-CD98hc KO cells (Fig. 5c). The replicative stress triggered by BCAA and AAA shortage was also recovered after addition of exogenous nucleosides, since the DDR was reversed in low 6AA cells (Supplementary Fig. S9). On the basis of our observations, we conclude that a shortage of nucleotides jeopardises faithful DNA replication in CD98hc KO cells, resulting in replicative stress and cell cycle arrest.

## Discussion

All cells take up nutrients from the surrounding environment into metabolic pathways in order to fuel the wide variety of functions that they exert^69^. However, proliferating cells, including cancer cells, have an increased nutritional demand compared to normal cells since they must double their biomass in each cell cycle. In this regard, AA transporters play a key role in meeting this metabolic challenge^70–72^. Our results highlight that CD98hc functions as a regulatory hub, orchestrating, not only AA availability and redox homeostasis, but also glucose and nucleotide metabolism. In this regard, we used low 6AA cells as a novel model through which to study the impact of BCAA and AAA shortage on protein synthesis and cell cycle regulation, independently of oxidative stress and other metabolic alterations present in CD98hc KO cells.

**Figure 6:**
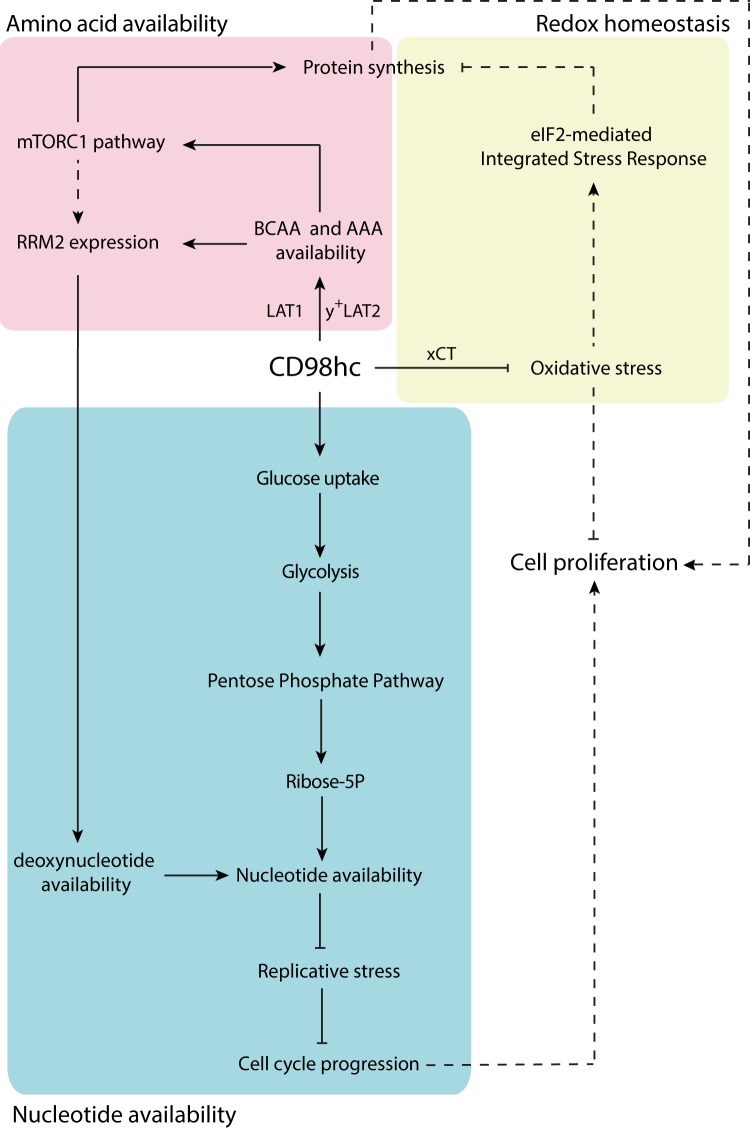
CD98hc sustains cellular nutrition, redox homeostasis and nucleotide availability, all key for cell proliferation. CD98hc-xCT is required for the counterbalance of the oxidative stress, thereby avoiding the activation of the eIF2α-mediated integrated stress response pathway. In addition, CD98hc sustains BCAA and AAA availability, mostly mediated via LAT1, although contribution of y^+^LAT2 cannot be discarded, for general protein synthesis and cell proliferation, as evidenced by the downregulated mTORC1 activity, protein synthesis and proliferation rate in both cellular models CD98hc KO and low 6AA cells. Furthermore, as demonstrated in low 6AA cells, AA availability sustains RRM2 expression, and, as a consequence, has an impact in the reduction of ribonucleotides to the corresponding deoxynucleotides, thereby balancing the cellular nucleotide content. In the same line, CD98hc regulates the cellular nucleotide pool, likely through the regulation of the pentose phosphate pathway flux, which enables cells to progress adequately throughout the cell cycle. The lack of CD98hc triggers a reduction in the glucose uptake and disposal, resulting in repressed glycolysis, which probably underlies the pentose phosphate pathway abrogation and the subsequent DNA replicative stress. Solid lines represent established connexions proposed in this work. Dashed lines represent connexions suggested by the data provided herein and literature.

Several stress-response mechanisms, including the inhibition of the proliferation rate and the attenuation of protein synthesis, allow cells to adapt to CD98hc ablation. In this respect, we identified alterations in both mTORC1 and eIF2 nutrient- and stress-sensing pathways, both of which regulate protein synthesis and proliferation (Fig. 6). Our results indicate that mTORC1 downregulation is driven by BCAA and AAA shortage, in agreement with previously reported results (Fig. 6)^8,19,36,73–75^. In support of this notion, the addition of BCAA- and AAA-containing dipeptides partially rescues cell proliferation^13^ and mTORC1 activity in CD98hc KO cells. Furthermore, low 6AA cells present similar mTORC1 pathway inhibition to that observed on CD98hc KO cells.

In contrast, we show that BCAA and AAA limitation does not induce the phosphorylation of eIF2α in our model. This finding is supported by the observation that the supplementation with dipeptides as an alternative source of BCAAs and AAAs does not ameliorate the activation of eIF2α in CD98hc KO cells. It is well established that, in conditions of AA starvation, uncharged tRNAs activate GCN2^76,77^, which in turn phosphorylates eIF2α^78–80^. However, to the best of our knowledge, neither tRNA charging, nor direct activation of GCN2 has been evaluated after partial AA restriction rather than full deprivation. Our results show that BCAA and AAA restriction to levels corresponding to the minimal physiological range in plasma is not enough to cause a substantial increase in the level of deacylated tRNAs. Thus, our data suggest that GCN2 does not govern the activated eIF2-mediated integrated stress response pathway in the CD98hc KO model, although its participation in the response cannot be ruled out. In this regard, a reduction of BCAAs and AAAs in WT cells (low 6AA cells) did not trigger any detectable change in the activity of eIF2α, despite the impaired proliferation rate existing in these cells. An alternative explanation for the observed activation of the integrated stress response could be that increased levels of oxidative stress in CD98hc KO cells mediate eIF2α phosphorylation (Fig. 6) through activation of the kinase heme-regulated inhibitor (HRI), which is stimulated in response to increased ROS levels^81–84^.

In keeping with these results, ablation of CD98hc leads to a reduced rate of protein synthesis, most likely due to repressed mTORC1, since low 6AA cells, which do not present induced eIF2α phosphorylation, show a similar impairment in global protein synthesis. Furthermore, this assumption is supported by the observation that the addition of BCAA- and AAA- containing dipeptides reversed cell proliferation defect in CD98hc KO cells^13^, while phosphorylation levels of eIF2α protein remained unaffected under these conditions.

In addition, other cellular processes, such as the cell cycle, are regulated by the nutritional status of the cell^85–87^. The progression of the cell cycle is tightly dependent on the ability of the cell to acquire nutrients and produce energy to drive the constant *de novo* biosynthesis of nucleotides^88,89^, which, when limiting, may affect genome integrity in diverse ways^90–92^. Our results indicate that CD98hc is essential for the biosynthesis of nucleotides (Fig. 6). Consistent with this, lack of CD98hc promotes a dramatic reduction in the nucleotide pool, which leads to ATR-CHK1-dependent DDR activation and subsequent protective arrest in S-phase. The induction of the DDR in CD98hc KO cells subjected to chronic replicative stress is likely to be governed by the overexpression and phosphorylation of the components of the pathway, probably as a result of a chronic adaptation^52–57^. Moreover, we report that a shortage of BCAAs and AAAs entails DNA damage and subsequent replicative stress in low 6AA cells. In this case, these cells present markedly increased phosphorylated levels of CHK1 and RPA, probably due to acute nutritional stress. Data provided by the transcriptome analysis suggest that the reduced mitotic rate in CD98hc KO cells is due to the activation of the G2/M checkpoint, which also involves the activation of CHK1^93,94^.

The addition of nucleosides rescued the replication-induced DNA damage in CD98hc KO cells, as reflected in the marked decrease of the phosphorylation of RPA and CHK1. Consistently, nucleoside-supplemented CD98hc KO cells partially resumed cycle progression, thus reversing the observed slowdown of the S-phase. Finally, the provision of nucleosides also rescued the slowed entry into mitosis. Collectively, these findings unveil that CD98hc is critical for the prevention of derangements in nucleotide levels.

The PPP is a major pathway for glucose metabolism and the main source of ribose-5P, which forms the sugar backbone of all nucleotides^95^. In this study, we propose that the abrogation of the PPP flux and, consequently, the drastic reduction of ribose-5P, explains the decreased levels of nucleotides in CD98hc KO cells (Fig. 6). In support of this notion, other studies have demonstrated that both ribose-5P scarcity^96,97^ and impaired PPP activity^98–101^ trigger compromised nucleotide biosynthesis and DNA replication.

CD98hc KO cells, in good agreement with the results obtained by Ohno *et al*., present downregulated levels of GLUT1, presumably as a consequence of its degradation caused by the absence of CD98hc^30^. In accordance, glucose uptake was reduced in these cells, probably leading to the dramatic repression of glycolysis observed in CD98hc-depleted cells (Fig. 6). Our results support the notion that the mechanism by which the PPP is downregulated lies in the alterations in glucose catabolism present in CD98hc null cells. In support of this hypothesis, glucose restriction has been previously linked to cell cycle arrest and nucleotide depletion^102–104^. In addition, we cannot discard the contribution of the decreased mRNA levels of G6PDH, probably triggered by mTORC1 downregulation^24^, to the supressed PPP.

On the basis of our data, we conclude that the mechanism by which the lack of CD98hc promotes a decrease in nucleotide levels is unlikely to be determined exclusively by the shortage of BCAAs and AAAs in CD98hc KO cells, since low 6AA cells did not present altered PPP activity. It has previously been reported that AA deprivation leads to a reduced production of phosphoribosyl pyrophosphate through the PPP and therefore decreased nucleotide synthesis^105–107^. However, in those studies, the authors performed full AA deprivation (arginine, isoleucine, leucine, lysine, tyrosine or valine), while low 6AA cells present a partial reduction in the levels of BCAAs and AAAs that is not sufficient to trigger the alteration of this pathway.

Low 6AA cells show a reduction only in the deoxynucleotide pool. The intracellular concentration of deoxynucleotides is tightly regulated by the expression of RRM2, the only enzyme that catalyses the rate-limiting step for the *de novo* conversion of ribonucleosides to deoxyribonucleosides^108,109^. Consistently, low 6AA cells presented a severe downregulation of RRM2 protein levels, which probably underlies the replicative stress reported in this model (Fig. 6). However, the link between BCAA and AAA restriction and RRM2 downregulation in low 6AA cells remains to be elucidated. In this regard, the ability of the oncogenic protein c-myc to control DNA replication is well established^110–112^. It has been reported that c-myc enhances the biosynthesis of nucleotides by promoting deoxynucleotide synthesis through the upregulation of RRM2^113,114^. Thus, it is feasible that low 6AA cells showed reduced c-myc expression. In support of this notion, Csibi and co-workers demonstrated that the mTORC1 pathway, which is downregulated in low 6AA cells, positively regulates c-myc translation^115^.

In summary, in this study we have evaluated the extensive cellular functions related to protein synthesis and cell cycle regulation that rely on CD98hc (Fig. 6). Our data further support CD98hc as a putative target in pathophysiological scenarios, especially in the context of cancer treatment. On the one hand, targeting CD98hc downregulates tumour growth by decreasing the redox counterbalance capacity mediated by CD98hc-xCT^116,117^, by limiting the required balance in the AA content for proper protein synthesis and cell proliferation, mostly harmonised by CD98hc-LAT1^118–122^, and by compromising integrin-regulated signalling pathways^4,5,13^. On the other hand, here we demonstrate that BCAAs and AAAs are required for deoxynucleotide synthesis. In addition, CD98hc sustains glucose uptake and metabolism, thereby regulating glycolysis and the PPP. Consequently, CD98hc ablation causes a broader alteration of nucleotide synthesis, resulting in replicative stress and cell cycle arrest. Such a therapeutic strategy would be beneficial for cancer treatment, as it would impair tumour cell proliferation. Moreover, the arrest in S-phase may enhance the sensitivity of cancer cells to chemotherapeutic^123,124^, radiotherapeutics^125–127^ agents, and to DDR inhibitors, as demonstrated here. Thus, combinatorial therapy involving the ablation of CD98hc emerges as a promising strategy for the treatment of cancer.

## Methods

### Cell culture

WT and CD98hc KO fibroblasts derived from mouse embryonic stem cells were generated by Chloé C. Féral (Université de Nice - Sophia Antipolis, Nice, France)^4^. WT and KO cells were cultured in DMEM high glucose (21969035, ThermoFisher) medium supplemented with 10% v/v FBS (SH30066.03, HyClone), 20 mM Hepes, pH 7.3, 100 μM non-essential amino acids (11140035, ThermoFisher), 2 mM L-glutamine (25030-024, ThermoFisher), 100 μM β-mercaptoethanol (31350010, ThermoFisher) and 100 U/mL Penicillin-Streptomycin (15140122, ThermoFisher).

WT cells named as control and low 6AA cells were cultured for 3 days before performing the corresponding experiments in DMEM medium (D9800-13, Stratech) supplemented with 10% v/v FBS (SH30066.03, HyClone), 20 mM Hepes, pH 7.3, 100 μM non-essential amino acids (11140035, ThermoFisher), 2 mM l-glutamine (25030-024, ThermoFisher), 100 μM β-mercaptoethanol (31350010, ThermoFisher), 100 U/mL Penicillin-Streptomycin (15140122, ThermoFisher), 25 mM glucose, 1 mM sodium pyruvate, 44 mM sodium bicarbonate and the same concentration of amino acids as complete DMEM media, with the exception of BCAAs and AAAs in low 6AA media (16 μM L-Isoleucine, 16 μM L-Leucine, 8 μM L- Phenylalanine, 1.5 μM L-Tryptophan, 7.8 μM L-Tyrosine and 16 μM L-Valine). Total AA concentrations are indicated in Supplementary Fig. S1a. Cells were maintained at 37 °C and 5% v/v CO_2_ in a humidified incubator and were periodically tested with a PCR detection kit to ensure that they were mycoplasma-free (MP0035, Sigma-Aldrich).

### Dipeptide and nucleoside supplementation

BCAA- and AAA-containing dipeptides were synthesised by the Synthesis of Peptides Unit (U3) of the CIBER in Bioengineering, Biomaterials and Nanomedicine (CIBER-BBN) at the Barcelona Science Park, as reported in^13^. Where indicated, dipeptides were supplemented in complete DMEM medium. BCAA: isoleucine (200 µM), leucine (200 µM), and valine (25 µM), or AAA: phenylalanine (25 µM), tyrosine (200 µM), and tryptophan (100 µM).

Where indicated, nucleosides (ES-008-D, EmbryoMax) were supplemented for 48 h (150 µM cytidine, 150 µM guanosine, 150 µM uridine, 150 µM adenosine and 50 µM thymidine).

### Protein synthesis measurement

Cells were washed twice with Phosphate-Buffered-Saline (PBS) and incubated for 1 min at 37 °C with labelling media (Met/Cys-free DMEM with the usual supplements, dialysed FBS and 120 Ci/mL of ^35^S-Methionine/Cysteine (Perkin Elmer)). Next, cycloheximide (C4859, Sigma-Aldrich) was added for 1 min at 37 °C at a final concentration of 100 μg/mL. Cells were then washed three times with cold PBS (plus 5 mM Met and Cys). They were then harvested in 0.5 mL of cold PBS and pelleted by centrifugation for 3 min at 3000 *g* at 4 °C. Cells were lysed for 30 min at 4 °C with NET buffer (50 mM Tris HCl pH 7.4, 150 mM NaCl, 5 mM EDTA, 0.5% IGEPAL, with protease inhibitors) and, after centrifugation for 10 min at 10000 *g*, the supernatant was precipitated with cold 10% Trichloroacetic acid in order to measure the amount of intracellular radioactivity incorporated into protein. The percentage of intracellular radioactivity incorporated was calculated.

### Immunoblotting

Cells were lysed in radioimmunoprecipitation assay (RIPA) buffer (150 mM NaCl, 10 mM Tris, pH 7.2, 0.1% w/v SDS, 1% w/v Triton X-100, 1% w/v deoxycholate, 5 mM EDTA, phosphatase inhibitor cocktail (524628-1SET, Merck Chemicals & Life Science S.A.) and protease inhibitor mixture (11836153001, Sigma-Aldrich). After centrifugation at 10,000 *g* for 15 min at 4 °C, protein concentrations were determined using the Pierce BCA protein assay (23225, Cultek). When total membrane extraction was required, cells were rinsed in homogenization buffer: 25 mM Hepes, 4 mM EDTA, 250 mM sucrose, phosphatase inhibitor cocktail (524628-1SET, Merck Chemicals & Life Science S.A.) and protease inhibitor mixture (11836153001, Sigma-Aldrich). Cell extracts were disrupted by three x 30 sec rounds of sonication. Centrifugation was then performed at 10,000 g for 10 min at 4 °C, and supernatants were then centrifuged at 200,000 g for 1 h at 4 °C. Protein extracts (20 μg, unless specified otherwise) were separated by electrophoresis on 10% or 15% SDS polyacrylamide gel and transferred onto Immobilon membranes (IPVH00010, Millipore). Membranes were blocked in 5% non-fat milk or 3% BSA according to datasheet specifications and incubated with the anti-mouse antibodies indicated in Supplementary Table S1. Immunoreactive bands were detected with horseradish peroxidase anti-mouse (715-035-150, Jackson Immuno Research Europe, 1:10.000), anti-rabbit (711-035-152, Jackson Immuno Research Europe, 1:10.000), or anti-rat (SC-2956, Santa Cruz, 1:15.000) antibodies using the ECL system (RPN2106, Sigma-Aldrich). Analysis and quantification of immunoblotting were performed using ImageJ software. At least three independent experiments were performed in duplicates for each immunoblotting experiment, with the exception of those corresponding to RRM2 and GLUT1 proteins, for which four and six independent experiments, respectively, with single samples for each were carried out.

### Cell proliferation assay

Control and low 6AA cells were seeded in duplicate in corresponding media at 1 × 10^4^ cells per 35-mm diameter dish. Cells were collected and counted at indicated time points using the Neubauer chamber method.

### Viability assay upon AZD7762 treatment

WT and CD98hc KO cells were cultured for 24 h and then treated with AZD7762 (0.5, 2 and 5 µM) for another 24 h. Cell viability was assessed by FACS using propidium iodide.

### Annexin V/PI apoptosis staining

The annexin V–FITC (A23204, Invitrogen) and propidium iodide (PI) (P4864, Sigma-Aldrich) double staining technique was used to evaluate apoptosis. Cells were seeded in 6-well plates at a density of of 1 × 10^5^ cell/mL. A positive control was performed by treating cells with 1 µM of staurosporine (S5921, Sigma-Aldrich) for 3 h. Cells were then harvested, washed in PBS and centrifuged. They were resuspended in 100 µl of Annexin V binding buffer. Samples were incubated with 5 µL of annexin V conjugate and 1 µL of PI for 15 min at room temperature (RT). Then, 300 µL of binding buffer was added to each sample, which was then subjected to flow cytometry (Gallios Flow Cytometer, Beckman Coulter). FSC-Area (forward scatter) and SSC-Area (side scatter) gating was applied to discriminate single cell population from debris. FITC and PI fluorescence were detected at 515 nm and 620 nm respectively. Ten-thousand events were recorded for each sample. Fluorescence was displayed on a scatter plot with PI and FITC quadrant gates. Data acquisition was performed using FlowJO software.

### Cell cycle phase distribution analysis

Cells were seeded in 6-well plates at a density of 1 × 10^5^ cell/mL with no additives or in the presence of nucleosides. When indicated, cells were synchronised by double thymidine block^43,128^. In that case, cells were incubated with 2 mM of thymidine (T1895, Sigma-Aldrich) for 16 h. Next, they were incubated for 8 h in complete medium without thymidine, and another block (2 mM thymidine) was performed for 16 h. After the double block, cells were released in complete medium for the indicated times. For cell cycle analysis, the unattached cells were collected from the growth media and pooled before washing the culture with PBS. Next, PBS was pooled and cells were trypsinised. Cells were then fixed in 70% ethanol and stored at −20 °C for at least 2 h. Before the analysis, cells were centrifuged and resuspended in propidium iodide/Triton X-100 (9036-19-5, Merck) staining solution with RNase A (740505, Cultek) (0.1% (v/v) Triton X-100, 2 mg RNase, 500 µM PI) for 30 min at RT. Samples were then subjected to flow cytometry (Coulter EPICS (R) XL Flow Cytometry System). Forward and side scatter area gating were used to identify singlets. Interval gates were placed on the detected peaks corresponding to the phases of the cell cycle. Percentage of cells in G1-, S-, and G2/M-phases was determined using MCycle software. Cell cycle profiles were generated using FlowJO software.

### 5-Ethynyl-2′-deoxyuridine (EdU) incorporation and detection by flow cytometry

Cells were seeded in 6-well plates at a density of 1 × 10^5^ cell/mL and incubated with EdU (10 µM) for 1 h (Click-iT™ Plus EdU Alexa Fluor™ 647 Flow Cytometry Assay Kit, Invitrogen). After washing with PBS three times, cells were cultured in fresh complete media for 2, 4 and 8 h. They were then washed with 1% BSA, fixed in 70% ethanol and stored at −20 °C for at least 2 h. Next, cells were washed again with 1% BSA and incubated with 1 × Click-iT™ saponin-based permeabilization and wash reagent for 15 min at room temperature. They were subsequently incubated with Click-iTEdU reaction buffer at room temperature for 30 min protected from light. For propidium iodide staining, cells were then resuspended in propidium iodide/Triton X-100 (9036-19-5, Merck) staining solution with RNase A (740505, Cultek) (0.1% (v/v) Triton X-100, 2 mg RNase, 500 µM PI) for 30 min at room temperature. Cells were then subjected to flow cytometry (Coulter EPICS (R) XL Flow Cytometry System). Forward and side scatter area gating were used to identify singlets. EdU incorporation was detected using 633/635 nm excitation with a red emission filter (660/20 nm). The percentages of cells in each cell cycle phase were determined using FlowJO software.

### Quantification of intracellular ROS levels by dichlorofluorescein assay

The levels of intracellular free radicals were assayed by measuring intracellular oxidation of H_2_DCFDA. Cells were seeded onto 6-well plates in corresponding media in normal conditions. Cultures were incubated with 1 μM non-fluorescent H_2_DCFDA (C6827, Thermo Fisher). After a 30-min incubation, H_2_DCFDA is converted to highly fluorescent 2′, 7′-dichlorofluorescein (DCF) upon cleavage of the acetate groups by intracellular esterases and oxidation. Cells were then harvested and washed in PBS, and intracellular fluorescence was measured using the Gallios Flow Cytometer system (Beckman Coulter).

### Gene expression analysis

Total RNA from the cell culture was extracted using an RNA extraction kit (12183018 A, PURELINK RNA MINI KIT, Invitrogen) following the manufacturer’s instructions. RNA was reverse-transcribed with the reverse transcriptase SuperScript RTII (18064014, Invitrogen). Quantitative real-time PCR was performed using the ABI Prism 7900 HT real-time PCR machine (Applied Biosystems) and the SYBR Green PCR Master Mix (4368702, Thermofisher). The sets of specific primers specified in Supplementary Table S2 were used.

### RNA expression profiling

Total RNA from the cell culture was extracted using an RNA extraction kit (12183018 A, PURELINK RNA MINI KIT, Invitrogen) following the manufacturer’s instructions. RNA integrity was assessed using RNA Nano Assay (Agilent Bioanalyzer 2100) and RNA quantification was executed using Nanodrop ND 1000 Spectrophotometer. cDNA library preparation and amplification were performed from 25 ng total RNA using WTA2 (Sigma-Aldrich) with 17 cycles of amplification. 8 µg of cDNA was subsequently fragmented by DNAseI and biotinylated by terminal transferase obtained from GeneChip Mapping 250 k Nsp Assay Kit (Affymetrix). The hybridisation mixture was prepared following the Gene Atlas protocol (Affymetrix). Each sample target was hybridised to a Mouse Genome 430 PM array. After hybridisation for 16 h at 45 °C, washing and staining was performed in the GeneAtlas Fluidics Station (Affymetrix). The arrays were scanned in a GeneAtlas Imaging Station (Affymetrix). All processing was performed following the manufacturer’s recommendations. CEL files were generated from DAT files using Affymetrix Command Console software. To generate the log2 expression estimates, overall array intensity was normalised between arrays and the probe intensity of all probes in a probe set was summarised to a single value using the RMA (Robust Multichip Average) algorithm^129^. Microarray processing was performed at Functional Genomics Facility at IRB Barcelona.

### Bioinformatic analyses

Affymetrix arrays were normalized using RNA. Background correction and summarization^130^ as implemented in the “affyPLM” package^131^ from the R statistical framework^132^. Annotations for the HT-430 array version na34 were downloaded from Affymetrix (Affymetrix Analysis Center. Netaffx https://www.affymetrix.com/analysis/index.affx). A linear model was fitted in order to identify differentially expressed genes between conditions of interest with batch scan as covariate. The “lmFit” function from the “limma” package^133^ was used for fitting the model. Gene set enrichment analysis (GSEA), as implemented in^134^, was performed on all the genes in the array ranked by the t-statistic obtained from the model. For each gene, the t-statistic of the most variable probe was used as a representative. We also ran GSEA on custom gene sets.

### Immunofluorescence analysis of mitosis

Cells were fixed in 4% paraformaldehyde (PFA) (sc-281692, Santa Cruz) in PBS for 20 min and washed with 50 mM NH_4_Cl. They were then permeabilised and blocked in 0.1% triton X-100 (9036-19-5, Merck), 2% FBS (F7524, Sigma-Aldrich) in PBS for 10 min. The coverslips were incubated with anti-phospho-Histone (P-H3) antibody (06-570, Merck) diluted 1:100 in 2% FBS PBS for 30 min. They were then washed with PBS, incubated in secondary antibody diluted 1:400 in 2% FBS PBS for 30 min and washed again with 0.5% triton X-100 PBS. They were then washed with PBS, stained with Hoechst 33342 (H3570, Invitrogen) diluted 1:20.000 in PBS and then washed again with PBS. Coverslips were mounted on microscope slides with Flouromount (17984-25, Fisher Scientic). WideField images were obtained using Olympus IX 81 microscope with objective lenses of 20×/0.45 LUCPlanFL N and 40x/0.75 UPlan FL N and ScanR Acquisition Software v2.3. Nuclei segmentation was performed using a tailor-made ImageJ macro. Mitosis was measured manually. Image processing and quantification were performed using ImageJ software.

### AA uptake measurement

Transport activities were studied on whole cells as previously described^135^ by measuring the transport of 10 µM L-[2,3-^3^H]-Arginine (American Radiolabeled Chemicals). To distinguish between y^+^ and y^+^L transport systems, L-arginine uptake assays were performed in Na^+^ media in the absence or presence of 1 mM L-leucine.

### Metabolite extraction

Cells were cultured for 16 h in the presence of fully labelled glucose (^13^C6-glucose, Sigma-Aldrich). Media were also collected. Cell pellets were scrapped, collected and frozen. Briefly, the pellets were resuspended in 300 µl of cold acetonitrile:methanol:water (5:4:1, v-v:v) containing ^13^C-glycerol (5 µl/ml) as internal standard. 10 µl of ^13^C3-glycerol (150 µl/ml) was also added to 200 µl of medium. Metabolites from cells were extracted with three rounds of liquid N2 immersion and sonication, followed by 1 h in ice before centrifugation at 14,500 rpm (10 min at 4 °C). Samples of media were lyophilised and resuspended in 500 µl of cold acetonitrile:methanol:water (5:4:1, v-v:v). After vortexing, they were placed in ice for 1 h and centrifuged at 14,500 rpm (10 min at 4 °C). Metabolite extractions of cells and media were split in aliquots of 200 and 400 µL for GC-MS, respectively, and 50 µL for LC-MS analysis.

### Gas chromatography-mass spectrometry analysis

Samples were dried under a stream of N2 gas and lyophilised before chemical derivatisation with 40 µl methoxyamine in pyridine (30 µg/ml) for 45 min at 60 °C. Samples were also silylated using 25 µl N-methyl-N-trimethylsilyltrifluoroacetamide with 1% trimethylchlorosilane (Thermo Fisher Scientific) for 30 min at 60 °C to increase the volatility of metabolites.  A 7890 A GC system coupled to a 7000 QqQ mass spectrometer (Agilent Technologies) was used for isotopologue determination. Derivatised samples were injected (1 µl) into the gas chromatograph system with a split inlet (5:1) equipped with a J&W Scientific HP-5ms stationary phase column (30 m × 0.25 mm i.d., 0.1 µm film, Agilent Technologies). Helium at a flow of 1.5 ml/min was used as carrier gas. The temperature gradient was from 70 to 190 °C at a heating rate of 11 °C/min and from 190 to 325 °C at 21 °C/min. Metabolites were ionized using positive chemical ionization (CI) with isobutene as reagent gas. Mass spectral data on the 7000 QqQ were acquired in scan mode monitoring ribose-5P (Retention time (min): 13.92; mass-to-charge ratio: 620), pyruvate (Retention time (min): 3.57; mass-to-charge ratio: 190) and lactate (Retention time (min): 3.72; mass-to-charge ratio: 235). The quantification of metabolites was based on peak areas; the indicated relative concentrations correspond to the peak area/cell number (Supplementary Dataset).

### Liquid chromatography-mass spectrometry analysis

To determine nucleotides, cell extracts were analysed using an UHPLC system coupled to a 6490 QqQ mass spectrometer (Agilent Technologies). Cell extracts were injected (5 µl) and metabolites were separated using an InfinityLab Poroshell 120 HILIC-Z column (2.7 µm, 2.1 × 100 mm, Agilent). The mobile phases used for the metabolite separation were A: 50 mM ammonium acetate with 5 µM medrionic acid; and B: acetonitrile. The chromatographic gradient was isocratic for 0.5 min at 80% B, from 0.5 to 7.5 min decreased to 70% B and from 7.5 to 8.5 min decreased to 50%, and maintained for 30 sec. From 9.0 min to 9.2 min the percentage of B rose quickly to 80% and finally the column was equilibrated at 80% B until min 11. Flow rate was 0.7 mL/min. The QqQ mass spectrometer worked in MRM mode using the transitions in Supplementary Table S3 to determine nucleotides. The electrospray ionization source (ESI) worked in positive and negative mode. The quantification of nucleotides was based on peak areas; the indicated relative concentrations correspond to the peak area/cell number (Supplementary Dataset).

### tRNA aminoacylation array

To determine the fraction of aminoacyl-tRNAs from the total tRNAs, we used tRNA-tailored microarrays and the protocol described earlier^136^. Total RNA was isolated using acidic phenol (pH 4.5) to preserve the aminoacyl moiety. The arrays were normalised to spike-standards, and quantification and normalisation was performed using in-house Phyton and R scripts.

### Intracellular AA quantification

Intracellular amino acid content was analysed using the Mass Trak Amino Acid Derivatization kit (186003836, Waters) and following the manufacturer’s instructions. Cells were collected and homogenised in water. 100 μL of 50 μM norvaline was added to 100 μL of each sample as an internal standard. Samples were vortex for 10 sec and centrifuged at 16,000 g for 5 min. Next, 20 μL of supernatant from each sample was mixed with 60 μL of NaOH 0.5 M/Borate buffer in a chromatography injection vial. After vortexing for 10 sec, 20 μL of 6-aminoquinolyl-N-hydroxysuccinimidyl carbamate (AQC) solution was added to the vials for AA derivatisation. Samples were then vortexed for 20 sec and incubated for 1 min at room temperature followed by 10 min at 55 °C. Sample preparations were injected into an Ultra High Performance Liquid Chromatograph (Shimadzu) (injection volume: 1 μL). Chromatography was performed using MassTrak AAA columns (2.1 × 150 mm, 1.7 µm) (Waters). Solutions A and B were used as mobile phases (A: MassTrak AAA Eluent A Concentrate, diluted 1:10; B: MassTrak AAA Eluent B) and MassTrak standard gradient was used as provided in the kit. Detection was performed at 260 nm. AAs were quantified with Labsolutions software (Shimadzu).

### Statistical analysis

Comparison of group means was performed using linear models with or without random effects depending on the data. Linear models were fitted with the R^132^ function “lm” and mixed effects models with the “lmer” function of the lme4 R package^137^. Whenever necessary, experiment was included as a fixed effect covariable. For the mixed effect models technical replicate was taken as a random effect. All data was log transformed except for panel 3c. The correct model for each dataset was chosen as follows: a mixed effect model was used when the variance explained by the replicate was larger than zero. Technical replicates were collapsed through the mean before log transforming when a linear model was chosen. Experiment was included as a fixed covariable if the model was significantly improved (F-test p-value lower than 0.25). Figures 1d,e,h and S1c (left and right graphs), 3c (lower right panel) and 4 g were analysed with a linear model, while all other panels were analysed with mixed effects models. In figures S1c (left and middle graphs), 3b (upper graphs) and 4 g, experiment was included as a fixed effect. In figures, 1e, 1h, S1c (left and right graphs) and 3c (lower right panel), replicates were collapsed to one observation through the mean. If not aforementioned a Statistical significance was analysed using a two-tailed Student’s t-test. Absolute values of normalized data have been included in Supplementary Dataset.

## Supplementary information

Supplementary Material

Supplementary dataset

## Data Availability

The raw RNA expression array data that support the findings of this study have been deposited in GEO with the accession code GSE126781. The rest of the data generated or analysed during this study are included in this published article and its supplementary information files.
