# Supplementary material for: CD98hc (SLC3A2) sustains amino acid and nucleotide availability for cell cycle progression
Source: Sci Rep. 2019 Oct 1;9:14065. doi: 10.1038/s41598-019-50547-9 (PMC6773781; doi:10.1038/s41598-019-50547-9)
Supplement: Supplementary file 1 — Supplementary Material [file 41598_2019_50547_MOESM1_ESM.pdf]

# CD98hc (SLC3A2) sustains amino acid and nucleotide availability for cell cycle progression

Sara Cano-Crespo[1*], Josep Chillarón[2], Alexandra Junza[3,4], Gonzalo Fernández-Miranda[1], Judit García[4,5], Christine Polte[6], Laura R. de la Ballina[7,8], Zoya Ignatova[6], Óscar Yanes[3,4], Antonio Zorzano[1,9,10], Camille Stephan-Otto Attolini[1] and Manuel Palacin[1,4,10*].

[*]Corresponding authors

Sara Cano-Crespo, sara.cano@irbbarcelona.org
Josep Chillarón, jchillaron@ub.edu
Alexandra Junza, alexandra.junza@urv.cat
Gonzalo Fernández-Miranda, gonzalo.fernandez@irbbarcelona.org
Judit García, JUGARCIA@clinic.cat
Christine Polte, Christine.Polte@chemie.uni-hamburg.de
Laura R. de la Ballina, l.r.de.l.ballina@medisin.uio.no
Zoya Ignatova, zoya.ignatova@chemie.uni-hamburg.de
Óscar Yanes, oscar.yanes@urv.cat
Antonio Zorzano, antonio.zorzano@irbbarcelona.org
Camille Stephan-Otto Attolini, camille.stephan@irbbarcelona.org
Manuel Palacín, manuel.palacin@irbbarcelona.org

Supplementary Figure S1

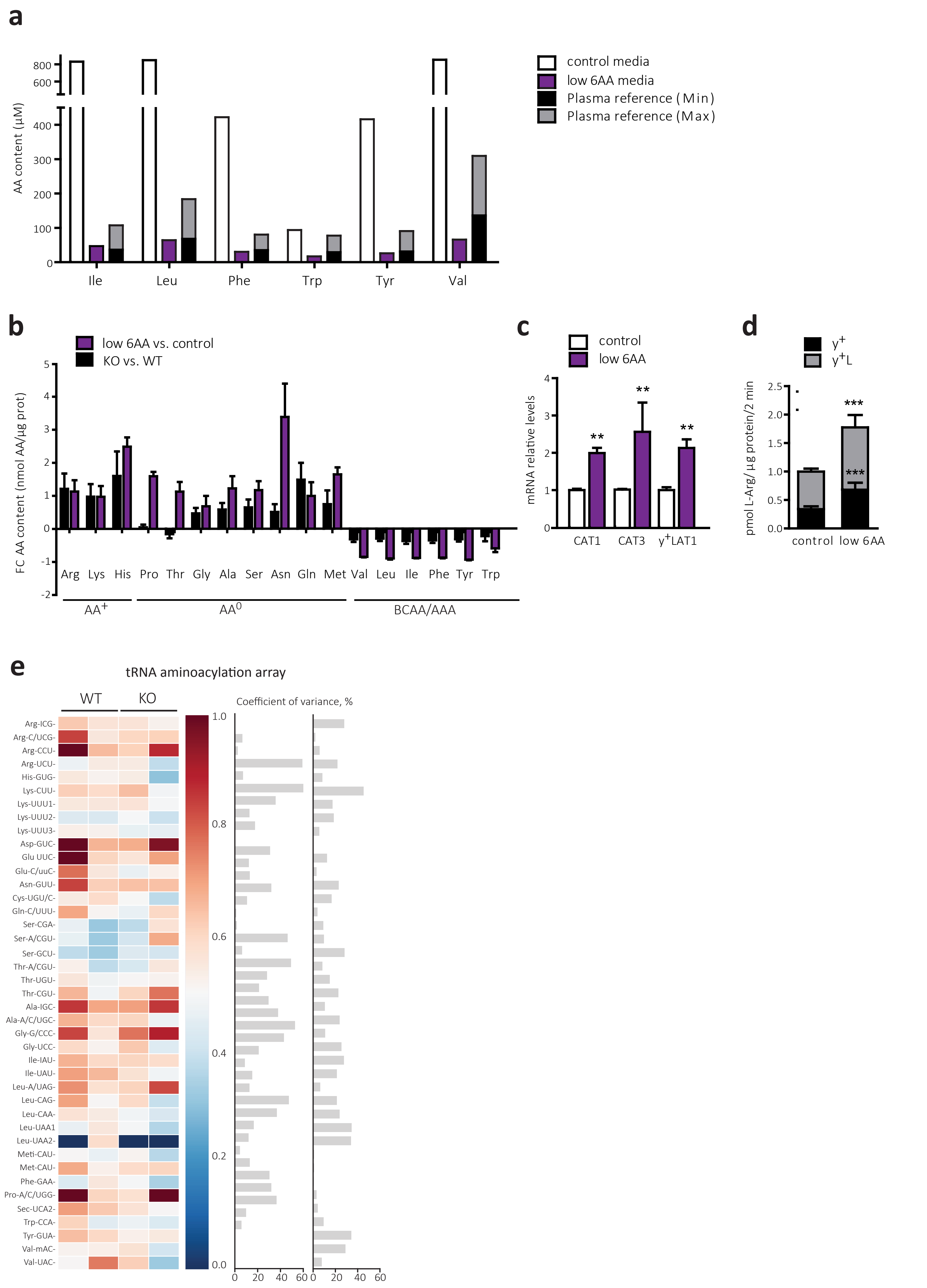

Supplementary Figure S1. Characterisation of cell adaptations to AA shortage

a, Final BCAA and AAA concentrations (µM) in control and low 6AA media are indicated and compared with minimum and maximum physiological reference values in human plasma.

b, Comparative quantitative analysis of the intracellular AA content (nmol AA/µg protein) in CD98hc KO (n=7) and low 6AA cells (n=5). The fold change (FC, (KO or low/WT or control)-1) of the AA concentration is shown. AAs are grouped by side chain properties as indicated.

c, Comparison of CAT1, CAT3 and $y^+$LAT1 mRNA expression levels between control and low 6AA cells. n=3.

d, L-arg (10 µM) uptake at 2 min (linear conditions of uptake) in control and low 6AA cells is indicated. L-Arg uptake inhibitable by 1 mM L-Leu + $Na^+$ was identified as system $y^+$L, the remaining transport was attributed to system $y^+$. n=4.

e, tRNA microarrays of aminoacyl-tRNAs in CD98hc KO and WT cells. Representative array of two independent biological replicates which are highly similar as assessed by covariance analysis (right plots) for each isoacceptor. tRNAs are depicted by their anticodon and cognate AA.

Data quantification correspond to the mean ± SEM of the independent experiments (n) indicated for each graph normalised to control or WT cells. Statistical significance *, $p \leq 0.05$; **, $p \leq 0.01$; ***, $p \leq 0.001$ vs. control or WT cells was analysed using a linear model (panel c) or a Student's t-test (panels d and e).

Supplementary Figure S2

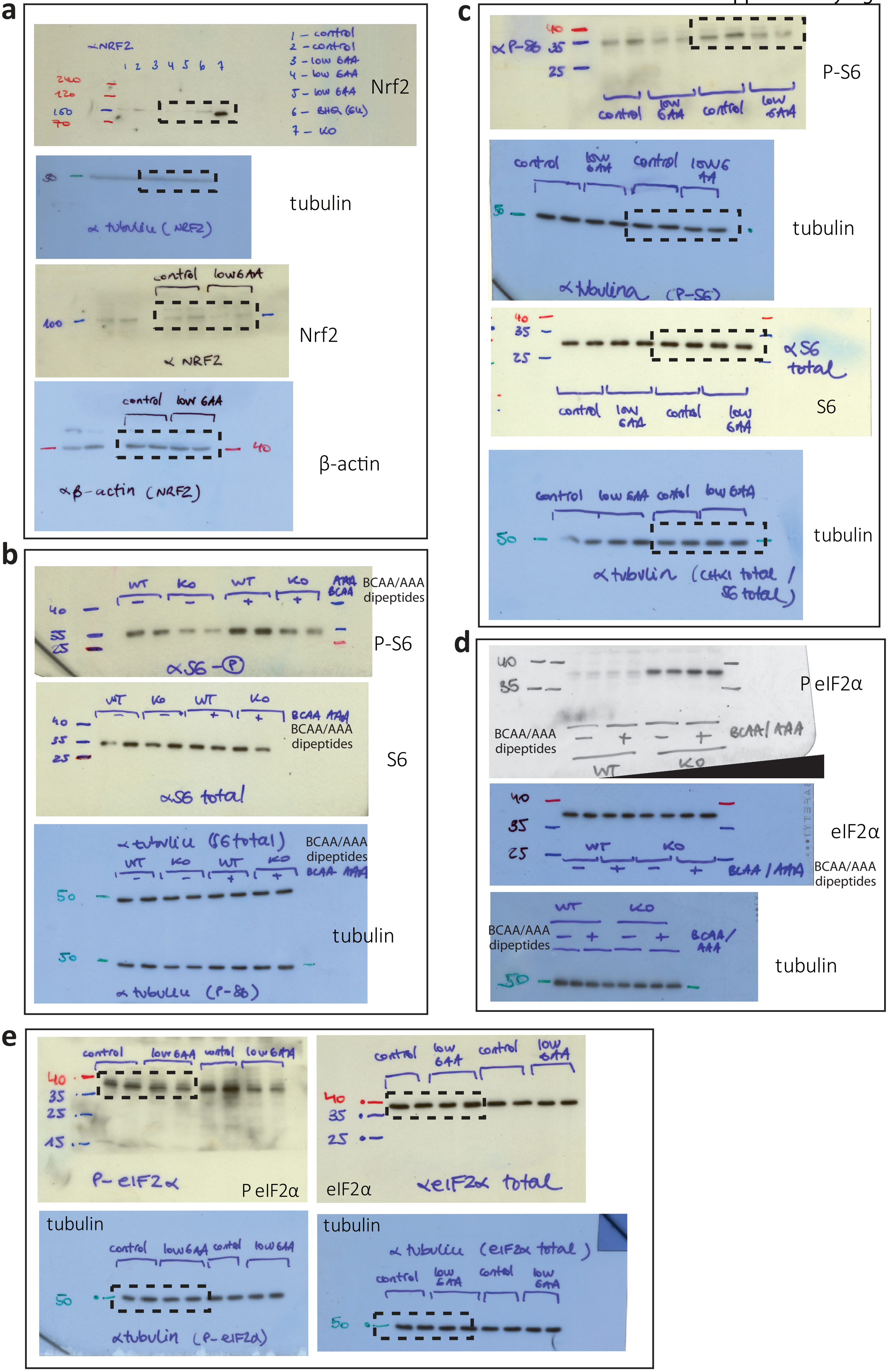

Supplementary Figure S2. Full-length blots corresponding to Figures:

**a**, 1d; **b**, 1e; **c**, 1f and 3c (lowest panel, tubulin corresponding to CHK1); **d**, 1g; **e**, 1h. The delimited areas correspond to the regions shown in the indicated figures.

Supplementary Figure S3

KO vs. WT

## Cell cycle related gene sets

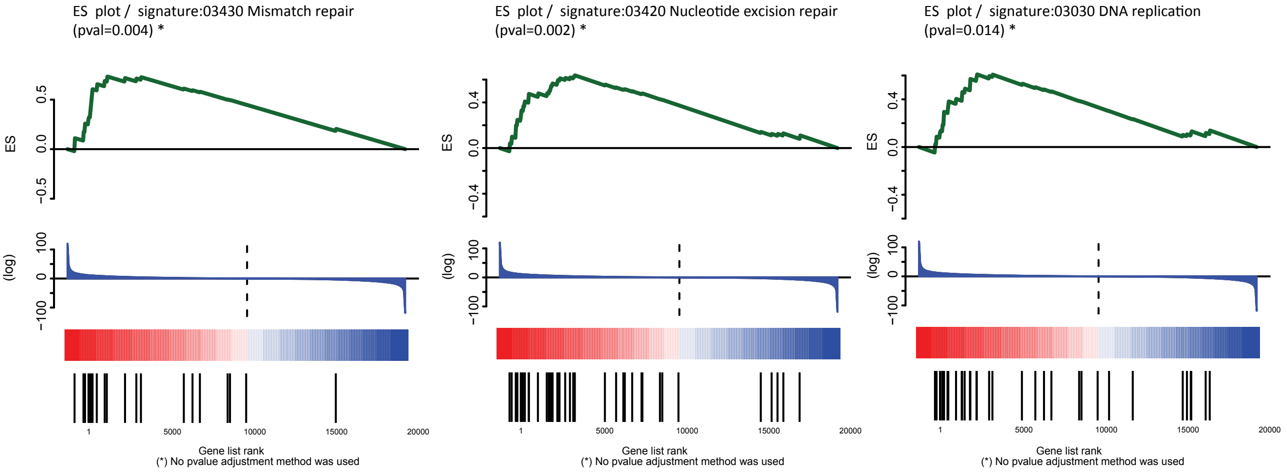


## Amino acid metabolism related gene sets

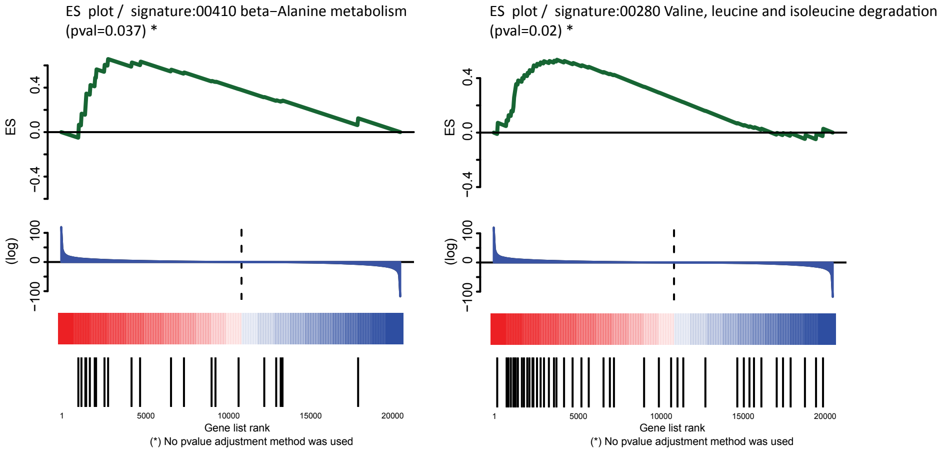


## RNA metabolism related gene sets

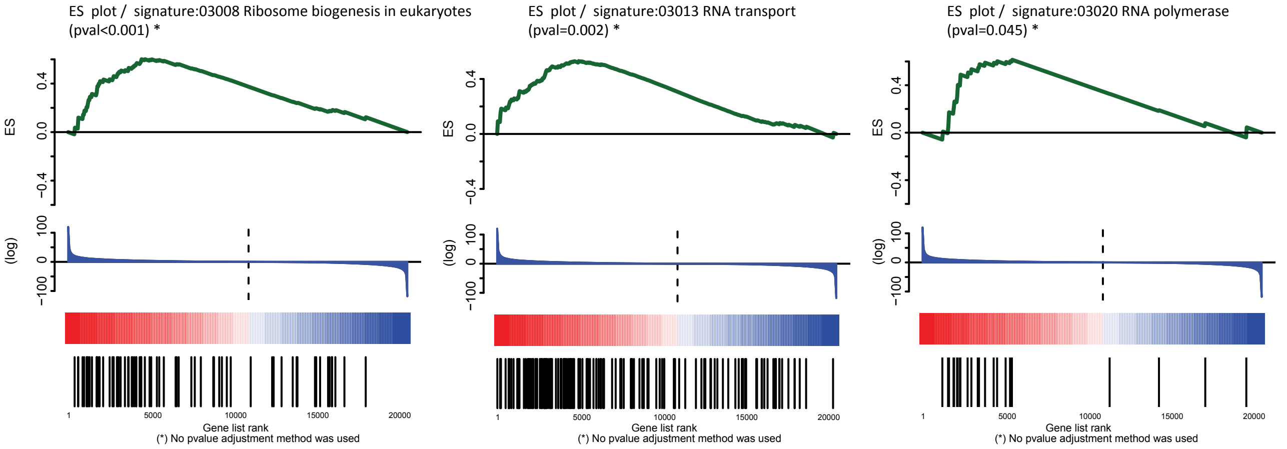


| Gene set | NES | p-val | FDR |
|---|---|---|---|
| 03008 RIBOSOME BIOGENESIS IN EUKARYOTES | 1.81 | 0.000 | 0.034 |
| 03430 MISMATCH REPAIR | 1.77 | 0.004 | 0.035 |
| 03013 RNA TRANSPORT | 1.76 | 0.002 | 0.025 |
| 03420 NUCLEOTIDE EXCISION REPAIR | 1.71 | 0.002 | 0.039 |
| 03030 DNA REPLICATION | 1.65 | 0.014 | 0.069 |
| 00410 BETA-ALANINE METABOLISM | 1.61 | 0.037 | 0.078 |
| 00280 VALINE, LEUCINE AND ISOLEUCINE DEGRADATION | 1.53 | 0.020 | 0.138 |
| 03020 RNA POLYMERASE | 1.52 | 0.045 | 0.130 |

**Supplementary Figure S3. GSEA reveals positive enrichment in gene sets related to cell cycle, AA metabolism and RNA metabolism in CD98hc KO cells**

Enrichment plots for the indicated gene sets obtained from gene set enrichment analysis (GSEA) using KEGG data base of transcriptional data from WT and CD98hc KO cells (upper panel). Y-axis: value of the enrichment score; X-axis: genes ranked by t-statistic. Bottom: plot of the ranked list of all genes. P-value (p-val), Normalized Enrichment Score (NES) and False Discovery Rate (FDR) are indicated for the included enrichment plots (lower panel).

Supplementary Figure 4

**a**

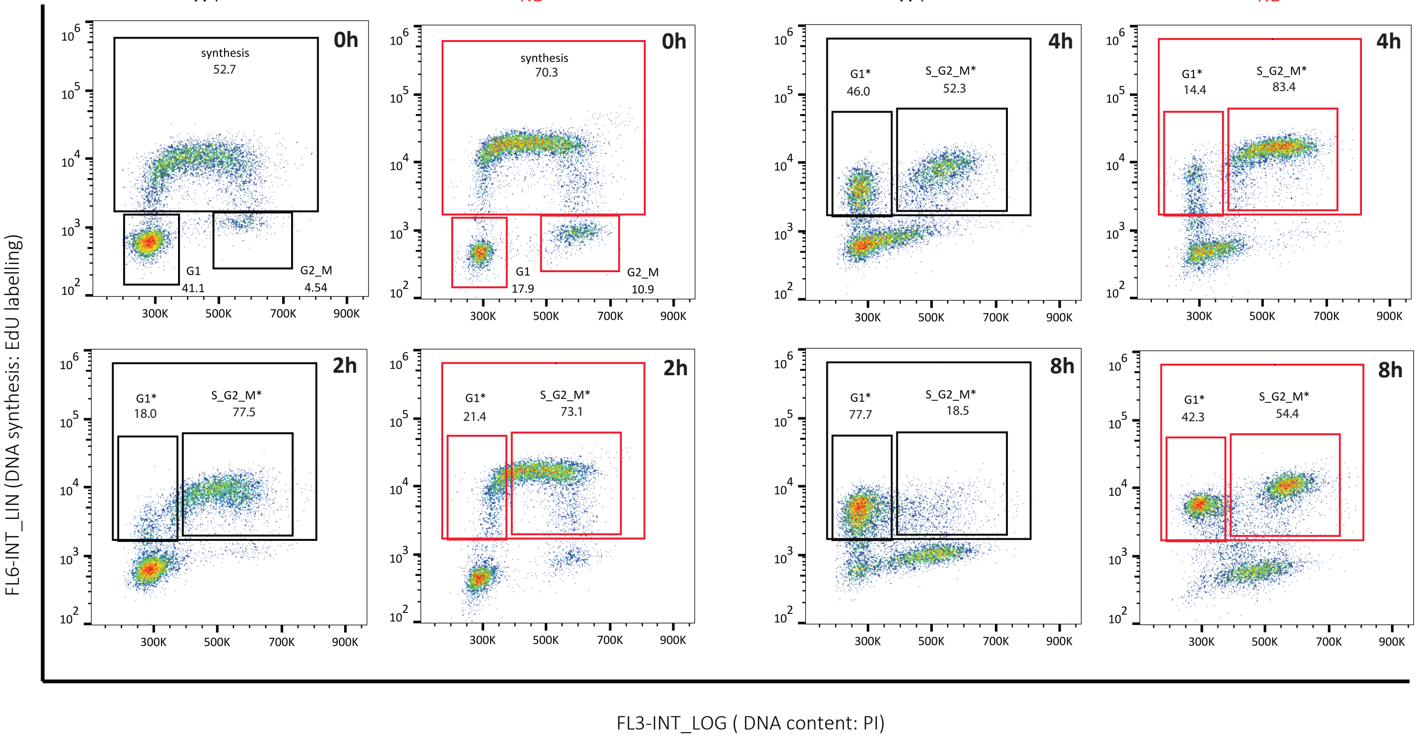


**b**

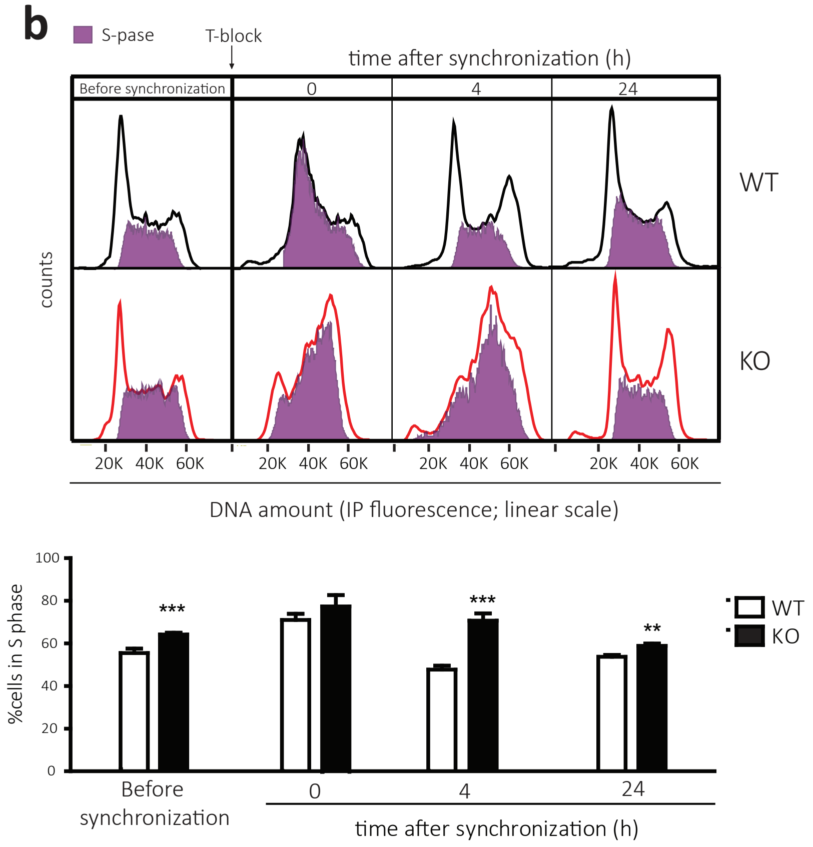

**Supplementary Figure S4. CD98hc depletion leads to delayed S-phase**

**a,** EdU pulse-chase time course showing the dynamics of actively dividing Edu-pulsed WT and CD98hc KO cells over 8 h. Cells were pulsed with 10 µM EdU for 1 h and stained with propidium iodide (PI) and fluorescent azide. EdU incorporation and PI staining were quantified by FACS. Representative FL3-DNA content vs FL6-EdU labelling contour plots displayed EdU pulsed WT and CD98hc KO cells for each time point are shown. Gates displaying the EdU labelled populations G1* and S_G2M * phases are shown. 10,000 cells/condition were analysed. The corresponding quantification from 3 independent experiments is shown in **Fig. 2c** and **Fig. 2d**.

**b**, WT and CD98hc KO cells were synchronised in S-phase with a double thymidine block as indicated in *Methods*. The DNA content was analysed by flow cytometry using propidium iodide (PI) staining. 10,000 cells/condition were analysed. The percentage of cells in each cell cycle stage was measured at the indicated times after the release of the thymidine block. A representative cell cycle profile with highlighted S-phase is shown for each time point (upper panel). The percentage of WT and CD98hc KO cells in S-phase out of total was quantified at the indicated time points for (lower panel). Data quantification correspond to the mean ± SEM of four independent experiments. Statistical significance **, $p \leq 0.01$; ***, $p \leq 0.001$ vs. WT cells was analysed using a Student's t-test.

Supplementary Figure S5

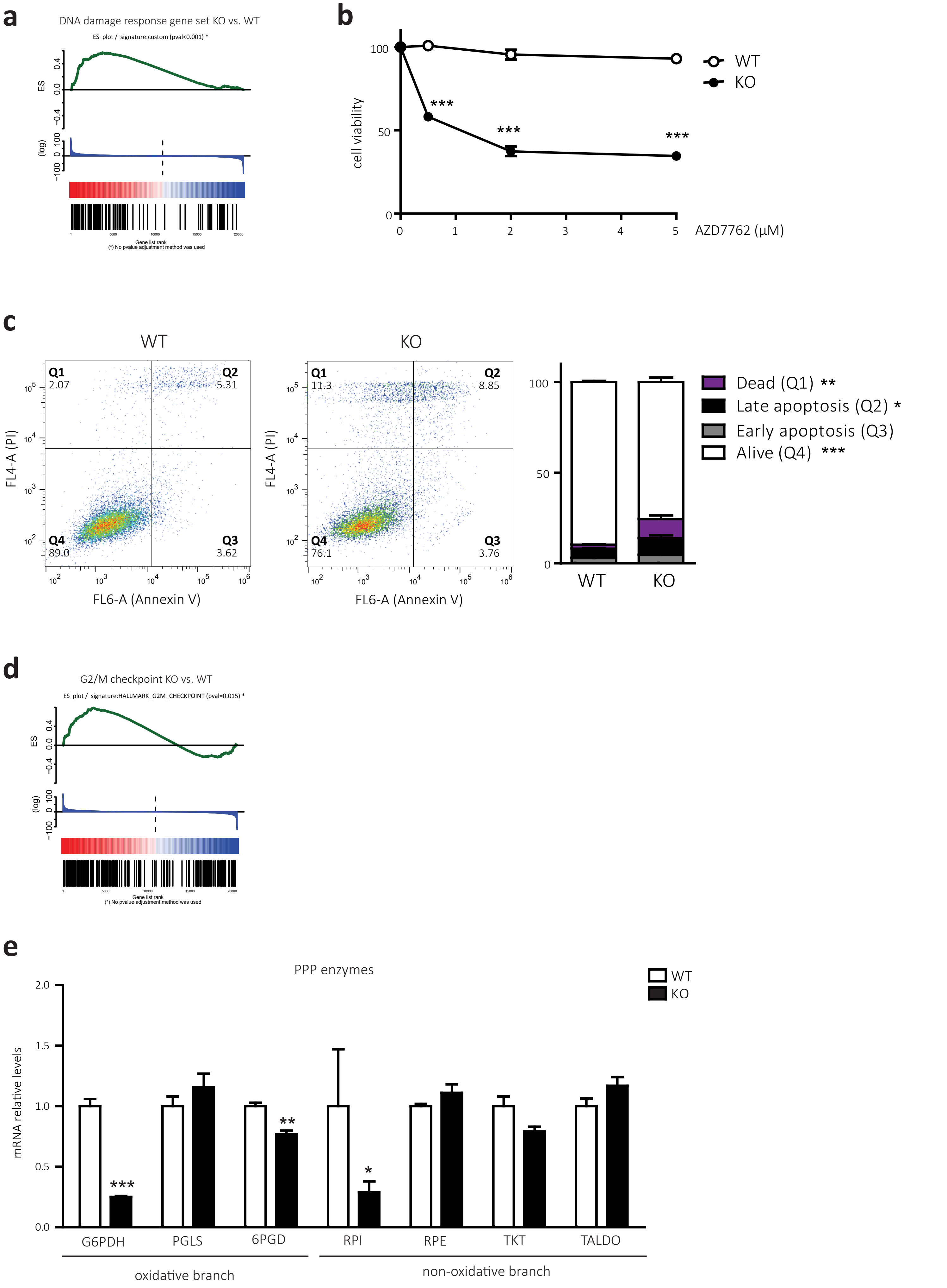

**Supplementary Figure S5. CD98hc KO cells activate the DNA Damage Response, induce apoptosis and have increased sensitivity to DNA replication stress inhibition.**

**a**, Enrichment plot for the gene set DNA Damage Response from gene set enrichment analysis (GSEA) of transcriptional data from WT and CD98hc KO cells. Y-axis: value of the enrichment score; X-axis: genes ranked by t-statistic. Bottom: plot of the ranked list of all genes. P-value=0.00, Normalized Enrichment Score (NES) =1.83 and False Discovery Rate (FDR) =0.00.

**b**, Cell viability of WT and CD98hc KO cells under AZD7762 treatment ( 0.5, 2 and 5 μM) for 24 h. Since in basal conditions CD98hc ablation leads to increased cell death (**Supplementary Fig. S5**) the effect of AZD7762 on cell viability was calculated over the total living cells without treatment. n=3.

**c**, Apoptosis in WT and CD98hc KO cells was measured by Annexin V and propidium iodide (PI) staining followed by flow cytometry analysis. Representative diagrams are shown (left panel). Y-axis: PI fluorescence; X-axis: Annexin V fluorescence. The analysis allows us to distinguish between living cells (lower left quadrant, Q4), early apoptotic cells (lower right quadrant, Q3), late apoptotic cells (upper right quadrant, Q2), and dead cells (upper left quadrant, Q1). The percentage of cells in each state was calculated and represented in a histogram (right panel). n=7.

**d**, Enrichment plot for the gene set G2/M checkpoint from gene set enrichment analysis (GSEA) using Hallmarks data base of transcriptional data from WT and CD98hc KO cells. Y-axis: value of the enrichment score; X-axis: genes ranked by t-statistic. Bottom: plot of the ranked list of all genes. P-value=0.015, Normalised Enrichment Score (NES) =1.37 and False Discovery Rate (FDR) =0.13.

**e**, G6PDH, PGLS, 6PGD, RPI, RPE, TKT and TALDO mRNA expression levels in WT and CD98hc KO cells. n=3.

Data quantification correspond to the mean ± SEM of the independent experiments (n) indicated for each graph. Statistical significance *, $p \leq 0.05$; **, $p \leq 0.01$; ***, $p \leq 0.001$ vs. WT was analysed using a Student's t-test.

Supplementary Figure S6

a

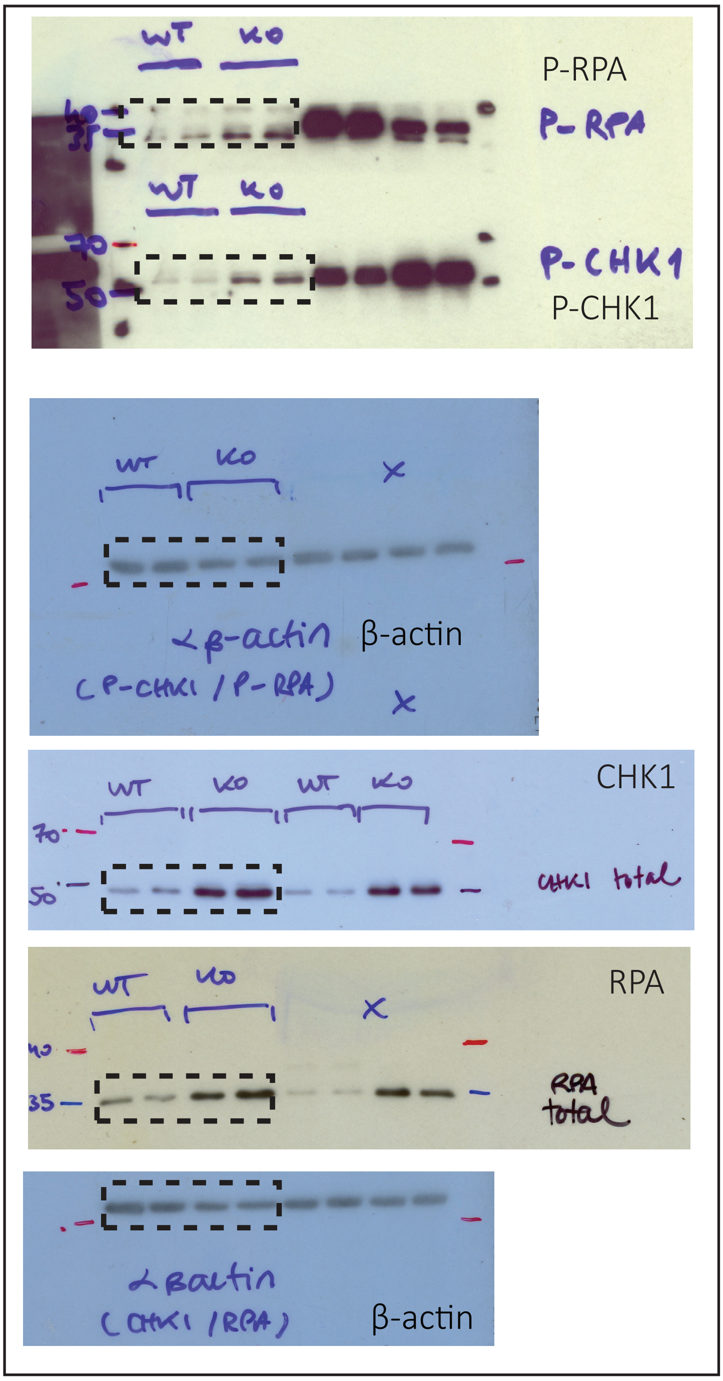


b

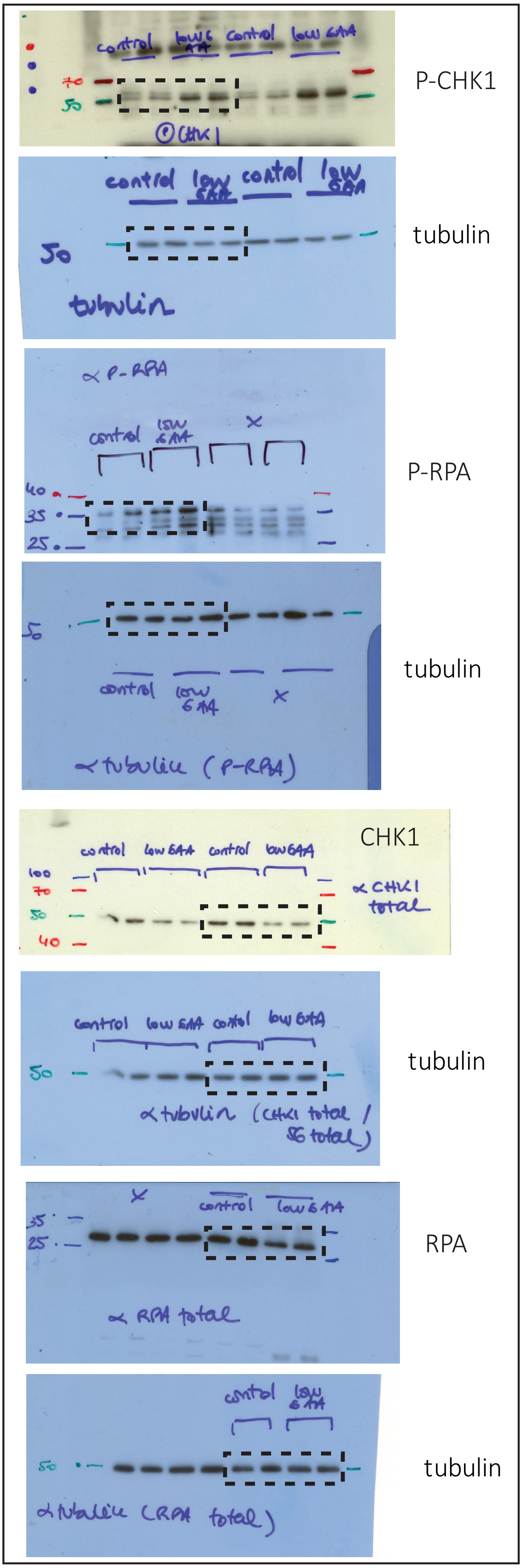

Supplementary Figure S6. Full-length blots corresponding to Figures:

a, 3b and b, 3c. The delimited areas correspond to the regions shown in the indicated figures.

Supplementary Figure S7

**a**

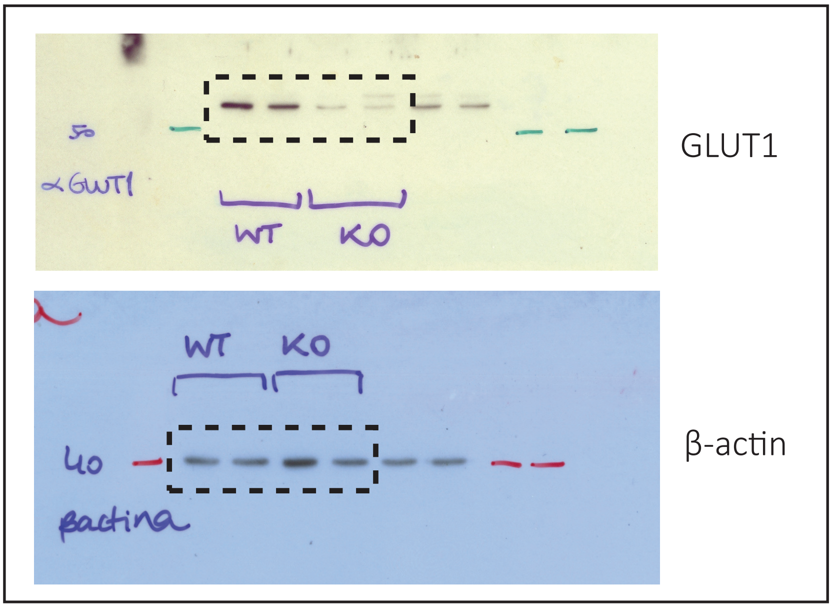


**b**

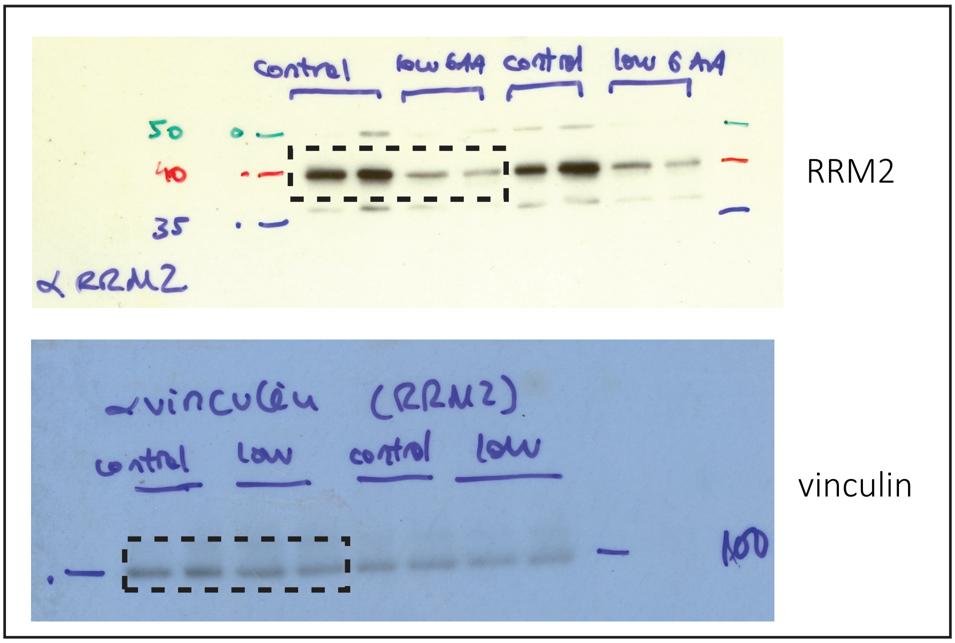

Supplementary Figure S7. Full-length blots corresponding to Figures:

**a**, 4c and **b**, 4g. The delimited areas correspond to the regions shown in the indicated figures.

Supplementary Figure S8

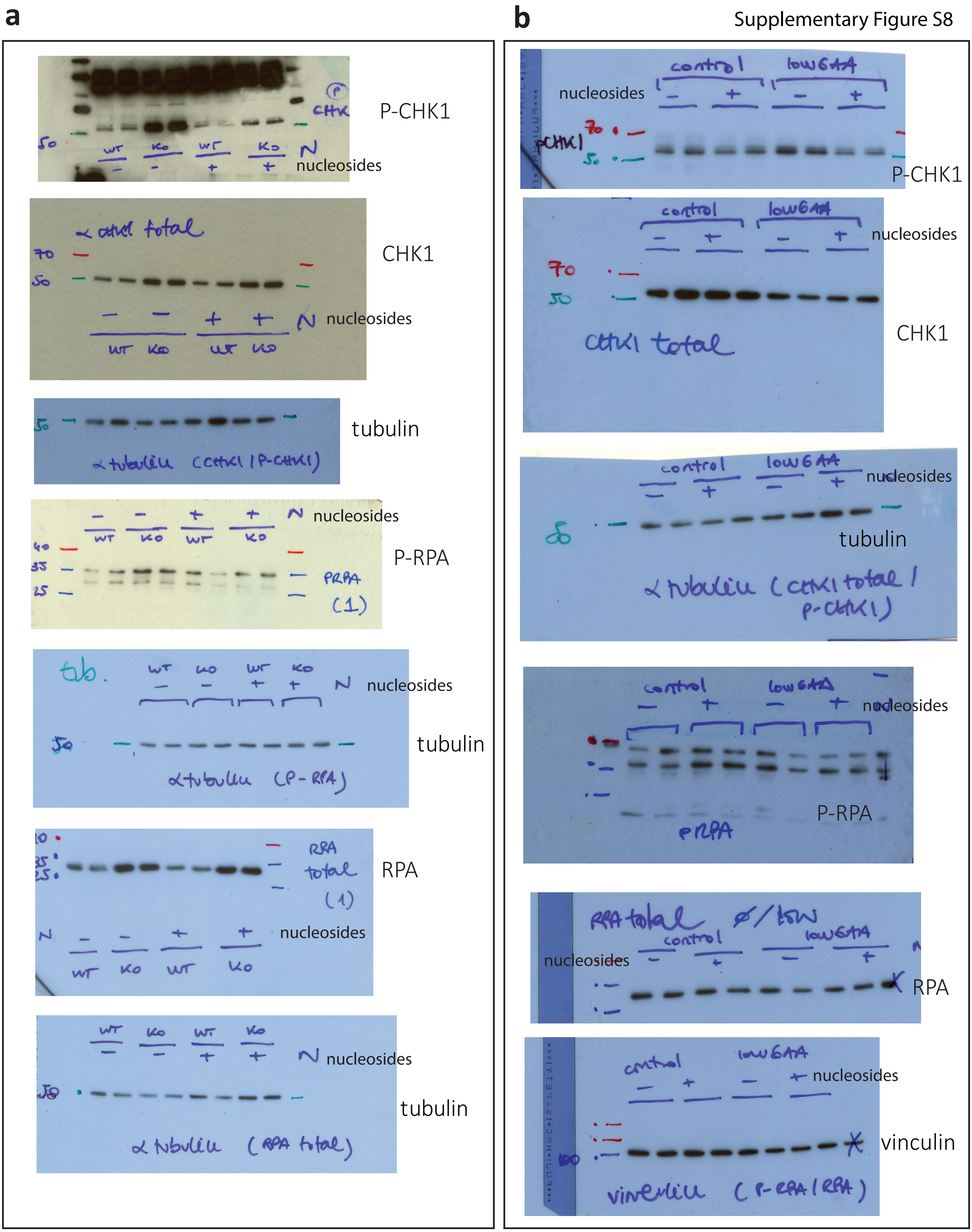

Supplementary Figure S8. Full-length blots corresponding to Figures:

**a**, 5b and **b**, S9.

# Supplementary Figure S9

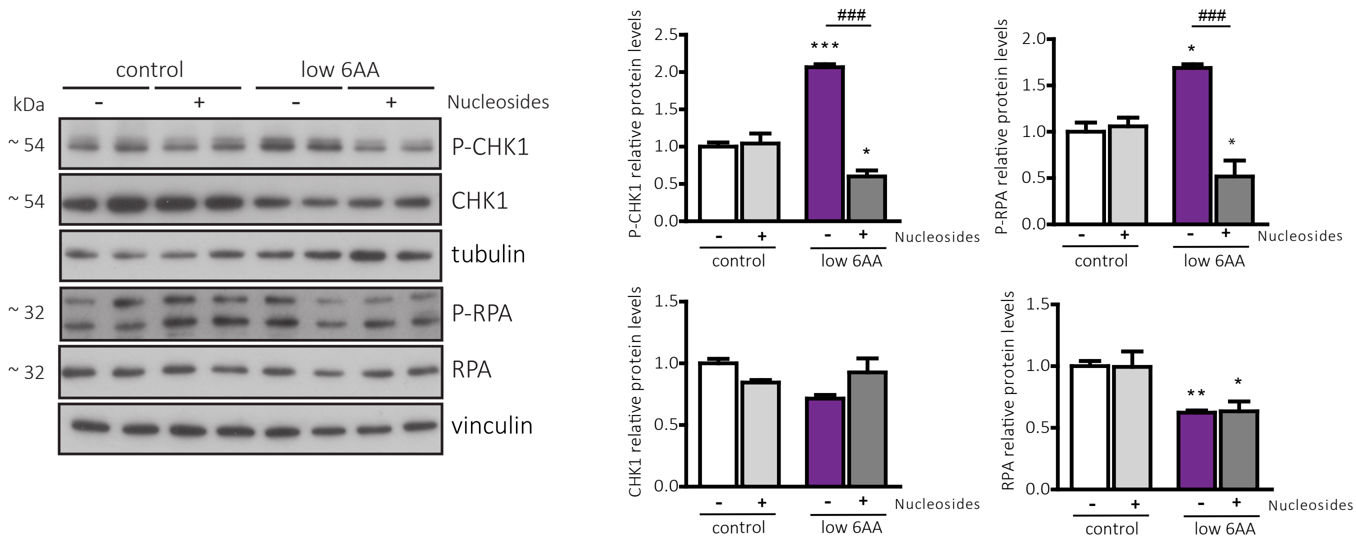

Supplementary Figure S9. Nucleosides reverse the replicative stress caused by BCAA and AAA deprivation in low 6AA cells

Comparison of phosphorylated and total protein levels of CHK1 and RPA between control and low 6AA cells with no additives ($n \geq 6$) or in the presence of nucleosides (48 h) ($n=4$). Data are normalised by tubulin or vinculin expression. Full-length blots are presented in **Fig. S8.** Quantification data correspond to the mean ± SEM of the independent experiments (n) indicated for each graph normalised to control cells. Statistical significance *, $p \leq 0.05$; **, $p \leq 0.01$; ***, $p \leq 0.001$ vs. control or #, $p \leq 0.05$; ##, $p \leq 0.01$; ###, $p \leq 0.001$ vs. low 6AA cells was analysed using a linear model.

Table S1. Antibodies used and details of usage

| Target | Working conditions | Cat. number | Source |
|---|---|---|---|
| P-S6 | 1:2000 | #2211S | Cell Signaling |
| S6 | 1:8000 | sc-74459 | Santa Cruz |
| P-eIF2α | 1:1000 | #9721 | Cell Signaling |
| eIF2α | 1:1000 | #9722 | Cell Signaling |
| P-CHK1 | 1:2000 | #2348 | Cell Signaling |
| CHK1 | 1:2000 | #2360 | Cell Signaling |
| P-RPA32 | 1:2000 | A300-245A-T | Bionova Cientifica |
| RPA32 | 1:2000 | 1672208T | S.G. Servicios Hospitalarios |
| Nrf2 | 1:1000 | ab62352 | Abcam |
| GLUT1 | 1:1000 | GT-11-A | Alpha Diagnostic |
| RRM2 | 1:1000 | GTX103193 | GeneTex |
| tubulin | 1:1000 | #T5168 | Sigma-Aldrich |
| Vinculin | 1:5000 | ab18058 | Abcam |
| β-Actin | 1:5000 | A1978 | Sigma-Aldrich |

Supplementary Table S2. Real time PCR SYBR Green primers

| Gene | F/R | 5'-3' sequence |
|---|---|---|
| Cationic amino acid transporter-1 (CAT1) | F | GGACACGGAGCGGAAAATACACC |
| | R | GCTCCCTGCTGTACTGGTTCATGGTC |
| Cationic amino acid transporter-3 (CAT3) | F | GGCTCCCTCTGTGCACTTTCTA |
| | R | TAGCAAGGACACGGAACAGGA |
| $y^+L$ amino acid transporter-1 ($y^+LAT1$) | F | CTAAAGGGCAATGCGAGCAAGCTG |
| | R | GGTGGTACCCAGTTCAGCGTAACAAAG |
| Ribosomal phosphoprotein, large, P0 (RPLP0) | F | CACTGGTCTAGGACCCGAGAAG |
| | R | GGTGCCTCTGGAGATTTTCG |
| Glucose-6-phosphate dehydrogenase (G6PDH) | F | CACAGTGGACGACATCCGAAA |
| | R | AGCTACATAGGAATTACGGGCAA |
| 6-Phosphogluconolactonase (PGLS) | F | CCAGGTCCTTACCATCAATCCT |
| | R | AGGGAAGAGCGAACAGGTATG |
| 6-Phosphogluconate dehydrogenase (6PGD) | F | TGAAGGGTCCTAAGGTGGTCC |
| | R | CCGCCATAATTGAGGGTCCAG |
| Ribose-5-phosphate isomerase (RPI) | F | AAGGCCGAGGAGGCTAAGAA |
| | R | CTTTCAGCTATTCGCTGCACA |
| Phosphopentose epimerase (RPE) | F | GCACCTGGATGTAATGGACGG |
| | R | CCTGGCCTAGCTGCTTTCG |
| Transketolase (TKT) | F | ATGGAAGGTTACCATAAGCCAGA |
| | R | TGCAGCATGATGTGGGGTG |
| Transaldolase (TALDO) | F | GTAAAGCGCCAGAGGATGGAG |
| | R | CTCTTGGTAGGCAGGCATCT |

F, forward; R, reverse

Supplementary Table S3. Characterization of analysed nucleotides.

| Metabolite | RT (min) | 1st Transition (CE (V)) | 2nd Transition (CE (V)) | Polarity |
|---|---|---|---|---|
| ATP | 5.67 | 508 ⟶ 136 (32) | 508 ⟶ 410 (12) | Positive |
| AMP | 3.13 | 348 ⟶136 (16) | 348 ⟶ 199(56) | Positive |
| ADP | 4.64 | 428 ⟶ 136 (32) | 428 ⟶ 348 (16) | Positive |
| dAMP | 2.79 | 332 ⟶ 136 (8) | 332 ⟶ 81 (12) | Positive |
| dADP | 4.34 | 412 ⟶ 136 (20) | 412 ⟶ 81 (36) | Positive |
| CMP | 4.40 | 324 ⟶ 112 (8) | 324 ⟶ 95 (48) | Positive |
| CDP | 5.89 | 404 ⟶ 112 (12) | 404 ⟶ 95 (56) | Positive |
| dCMP | 3.92 | 308 ⟶ 112 (12) | 308 ⟶ 95 (56) | Positive |
| dCDP | 5.51 | 388 ⟶ 112 (12) | 388 ⟶ 81 (20) | Positive |
| GMP | 4.46 | 364 ⟶ 152 (8) | 364 ⟶ 135 (52) | Positive |
| GDP | 5.88 | 444 ⟶ 152 (32) | 444 ⟶ 135 (60) | Positive |
| dGMP | 4.01 | 348 ⟶ 152 (8) | 348 ⟶ 135 (56) | Positive |
| UMP | 3.19 | 325 ⟶ 97 (8) | 325 ⟶ 41 (48) | Positive |
| UDP | 4.67 | 403 ⟶ 159 (24) | 403 ⟶ 79 (52) | Negative |
| dTDP | 3.89 | 401 ⟶ 79 (60) | 401 ⟶ 159 (24) | Negative |
| IMP | 3.76 | 349 ⟶ 137 (16) | 349 ⟶ 110 (60) | Positive |

RT, retention time; CE, collision energy; V, volts.
